# Connecting cilium, stress response, and proteostasis abnormalities inform variant and therapy assessment in *RPGRIP1* retinal organoids

**DOI:** 10.1016/j.stemcr.2025.102717

**Published:** 2025-11-20

**Authors:** To Ha Loi, Anson Cheng, Hani Jieun Kim, Milan Fernando, Benjamin M. Nash, Nader Aryamanesh, John R. Grigg, Pengyi Yang, Anai Gonzalez-Cordero, Robyn V. Jamieson

**Affiliations:** 1 Eye Genetics Research Unit, Children’s Medical Research Institute, Sydney Children’s Hospitals Network, Save Sight Institute, University of Sydney, Sydney, NSW, Australia; 2 Computational Systems Biology Unit, Children’s Medical Research Institute, University of Sydney, Sydney, NSW, Australia; 3 Stem Cell Medicine Group and Stem Cell and Organoid Facility, Children’s Medical Research Institute, University of Sydney, Sydney, NSW, Australia; 4 Sydney Genome Diagnostics, Western Sydney Genetics Program, Sydney Children’s Hospitals Network, Sydney, NSW, Australia; 5 Specialty of Genomic Medicine, Faculty of Medicine and Health, University of Sydney, Sydney, NSW, Australia; 6 Bioinformatics Facility, Children’s Medical Research Institute, Sydney, NSW, Australia; 7 Department of Ophthalmology, Sydney Children’s Hospitals Network, Sydney, NSW, Australia; 8 Swarbrick Laboratory, Garvan Institute of Medical Research, The Kinghorn Cancer Centre, Darlinghurst, NSW, Australia; 9 Department of Clinical Genetics, Western Sydney Genetics Program, Sydney Children’s Hospitals Network, Sydney, NSW, Australia

**Keywords:** RPGRIP1, retinal organoids, iPSC, ciliopathies, inherited retinal disease, photoreceptors, AAV therapy, gene augmentation, variants of uncertain significance, transcriptome

## Abstract

*RPGRIP1* encodes a connecting cilium (CC) protein essential for normal photoreceptor cell development and maintenance. Damaging variants in *RPGRIP1* cause severe inherited retinal disease (IRD) and currently incurable vision loss, with mouse studies showing promising preclinical gene augmentation therapy results. Almost one-half of variants in *RPGRIP1* in the ClinVar database are variants of uncertain significance (VUS), hindering genetic diagnosis for affected individuals and, hence, access to clinical trials of novel therapies and other management options. Here, we use human induced pluripotent stem cell (iPSC)-derived retinal organoids to model *RPGRIP1*-associated IRD, detecting biomarkers of disease including CC interactome dysfunction, stress response, and proteostasis abnormalities. In parallel, utilizing these novel disease biomarkers, we demonstrate the pathogenicity of a missense VUS, *RPGRIP1* c.2108T>C p.(Ile703Thr). In addition, *RPGRIP1* gene augmentation therapy rescued disease phenotypes, further supporting the utility of these biomarkers of *RPGRIP1*-IRD for reclassifying VUS and testing response to therapy.

## Highlights

• iPSC-retinal organoids recapitulate *RPGRIP1*-inherited retinal disease
• Biomarkers detect photoreceptor ciliary complex disruption in *RPGRIP1* organoids
• Bulk and single-cell RNA-seq reveal stress and retinal subpopulation signatures
• Biomarkers and signatures facilitate *RPGRIP1* variant and therapy assessment

## Teaser

In this article, Jamieson and colleagues describe the first 3D retinal organoid model for *RPGRIP1*-associated inherited retinal disease (IRD) from patient-derived and genome-engineered iPSCs. Key biomarkers identified disturbed protein interactions at the photoreceptor connecting cilium. Bulk and single-cell transcriptomics revealed stress and retinal subpopulation signatures. Proteostasis abnormalities and other biomarkers and signatures distinguished the *RPGRIP1* variant and therapy impact.

## Introduction

Leber congenital amaurosis (LCA) is a form of inherited retinal disease (IRD) that causes severe visual impairment in early childhood due to the abnormality or progressive degeneration of photoreceptor cells. Pathogenic variants in over 20 genes are known to cause LCA, including retinitis pigmentosa GTPase regulator-interacting protein (*RPGRIP1*) (OMIM: 605446), which accounts for 5%–7% of cases (Beryozkin et al., 2021; Kumaran et al., 2017). *RPGRIP1* encodes a ciliary protein expressed in the connecting cilium (CC) of both rod and cone photoreceptors cells. The CC connects the inner segment (IS) where protein is produced, with the outer segment (OS) region where light-sensing phototransduction occurs. RPGRIP1 anchors RPGR to the CC space between the axoneme and plasma membrane and serves as a structural/scaffolding protein that interacts with other ciliary proteins including CEP290 and NPHP4 (Gerner et al., 2010; Roepman et al., 2005). This protein interactome acts as a gate, controlling the trafficking of essential proteins such as light-sensing opsins, membranous structural proteins, and enzymes from the IS to the OS for proper disk morphogenesis, maintenance, and phototransduction (Patnaik et al., 2015).

There are over 1,000 variants in *RPGRIP1* in ClinVar (https://www.ncbi.nlm.nih.gov/clinvar; access: 17/1/2025) with a startling ∼47% classified as variants of uncertain significance (VUS). VUS impede the provision of a clinical genetic diagnosis and often prevent access to clinical gene therapies, clinical trials, and reproductive options. Structural and functional retinal clinical studies in patients with RPGRIP1-related IRD suggest that despite early-onset visual impairment, there may be relatively preserved retinal structure highlighting the potential value of gene augmentation therapy (Jacobson et al., 2007) and hence the need for resolution of VUS in *RPGRIP1*. The majority (84%) of *RPGRIP1* VUS are missense (MS) variants, which are particularly challenging to reclassify to a clinically useful pathogenic/likely pathogenic or benign/likely benign diagnosis (Richards et al., 2015). While mouse models of LCA resulting from *Rpgrip1* knockout offer some insight into the significance of RPGRIP1 in photoreceptor biology (Zhao et al., 2003), they have limited use for studying individual MS variants that lead to specific amino acid changes. Induced pluripotent stem cells (iPSC) differentiated to retinal organoids provide a renewable resource of human photoreceptor cells for modeling genetic variants in IRDs as demonstrated by ourselves and others (Chahine Karam et al., 2022; Kruczek et al., 2022).

Here, we used patient-derived and CRISPR-Cas9 genome-engineered iPSC-retinal organoids to establish a model system for biomarker detection and evaluation in *RPGRIP1* variant organoids, to facilitate reclassification of a missense VUS (MS-VUS) in *RPGRIP1* and test augmentation therapy. Retinal organoids with pathogenic variants in *RPGRIP1* demonstrated abnormalities of the CC interactome, opsin trafficking and photoreceptor development, and transcriptomic signatures of increased stress response and proteostasis abnormalities. Similar changes were also shown in the *RPGRIP1* VUS organoids, suggesting variant pathogenicity. Identified biomarkers were valuable in the assessment of adeno-associated viral (AAV) *RPGRIP1* gene therapy where the replenished expression of *RPGRIP1* in RPGRIP1-deficient organoids led to concurrent improvement in the identified biomarkers.

## Results

### *RPGRIP1*-LCA and identification of a novel *RPGRIP1* variant

We applied TruSight One Clinical Exome sequencing to examine a bioinformatic gene panel of 65 IRD genes, which identified *RPGRIP1* variants in 2 patients, LCA-1 and LCA-2, with poor vision and nystagmus manifested in the first 3 months of life. Electroretinograms showed reduced responses, and poor visual acuity was consistent at last review at ages 29 and 27 years for LCA-1 and LCA-2, respectively, with ophthalmic changes characteristic of LCA (Figures S1A and S1B).

**Figure 1:**
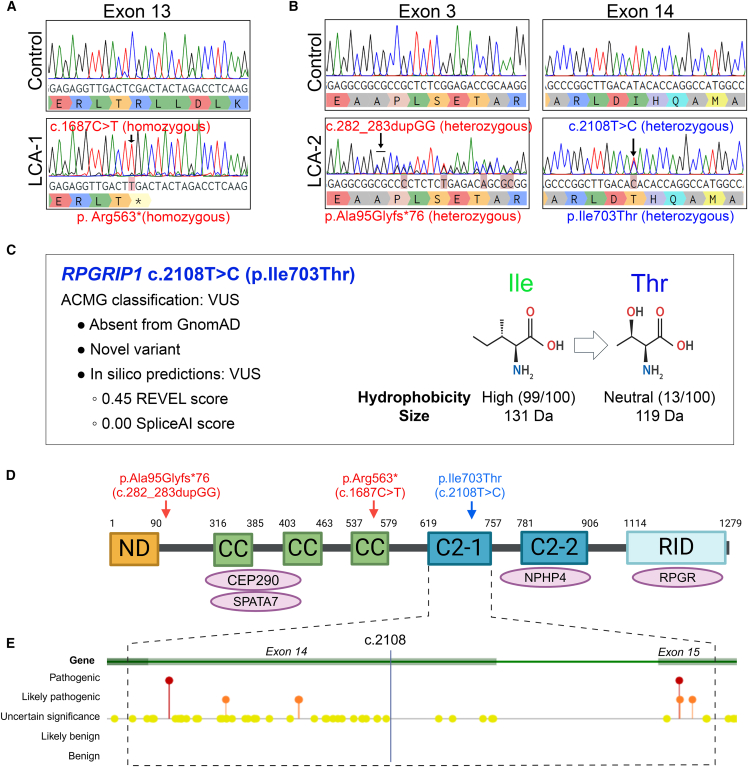
RPGRIP1 gene structure and variants examined in this study (A) Sanger sequencing showing the presence of *RPGRIP1* homozygous pathogenic variant c.1687C>T p.(Arg563^∗^) in LCA-1 genomic DNA. (B) Sanger sequencing showing the presence of *RPGRIP1* heterozygous variants (1) c.282_283dupGG p.(Ala95Glyfs^∗^76) pathogenic variant and (2) c.2108T>C p.(Ille703Thr) in LCA-2 genomic DNA. (C) Classification of the c.2108T>C variant as a VUS based on ACMG criteria. Amino acid hydrophobicity index at pH 7 is relative to glycine (value: 100), the most hydrophobic amino acid. (D) Schematic of the RPGRIP1 protein structure encoded by NM_020366.4. Key domains, protein-binding regions, and locations of the variants examined are shown. Variants in red are pathogenic. Created in https://BioRender.com. (E) Location of the MS variants listed in ClinVar within the first C2 domain (C2-1). 3 pathogenic variants (red dots); 5 likely pathogenic variants (orange dots); 46 VUS (yellow dots). Adapted from the ClinVar website.

LCA-1 had homozygous copies of a pathogenic nonsense variant in *RPGRIP1* (NM_020366.4): c.1687C>T p.(Arg563^∗^) (Figure 1A), creating a stop codon approximately halfway into the protein sequence with an expected loss-of-function phenotype. For LCA-2, two heterozygous *RPGRIP1* variants were identified (Figure 1B). The first was a pathogenic variant *RPGRIP1* (NM_020366.4): c.282_283dupGG p.(Ala95Glyfs^∗^76) inducing a predicted frameshift and premature stop codon early in the transcript. The second variant was a novel MS variant, *RPGRIP1* (NM_020366.4): c.2108T>C p.(Ile703Thr) that was classified as a VUS on the basis of American College of Medical Genetics and Genomics (ACMG) criteria (Richards et al., 2015) (Figure 1C). This variant was absent from the control population database, gnomAD, and was not reported in the literature or ClinVar. Aggregated assessment of *in silico* computational predictive tools resulted in a REVEL score of 0.45, which is outside the range for contribution to pathogenic or benign scoring (Pejaver et al., 2022). The SpliceAI score was 0.0, so there was no evidence to support a likely splicing effect. The variants in LCA-2 were unable to be segregated due to the unavailability of parental samples, so use of the in *trans* criterion for recessive variants (PM3 of the ACMG criteria) was unable to contribute to variant classification. However, it was noted that the c.2108T>C variant was in a region encoding a conserved domain in RPGRIP1, the first C2 domain (C2-1) (Figure 1D). Within the C2-1 domain are 8 MS variants classified as pathogenic or likely pathogenic in ClinVar (Figure 1E), although none with functional data. Furthermore, there are 48 other VUS of which 46 (96%) are MS variants, highlighting the need to determine the significance of the C2-1 domain in RPGRIP1 function and disease.

### *RPGRIP1* retinal organoids for disease modeling and variant phase evaluation

**Figure 2:**
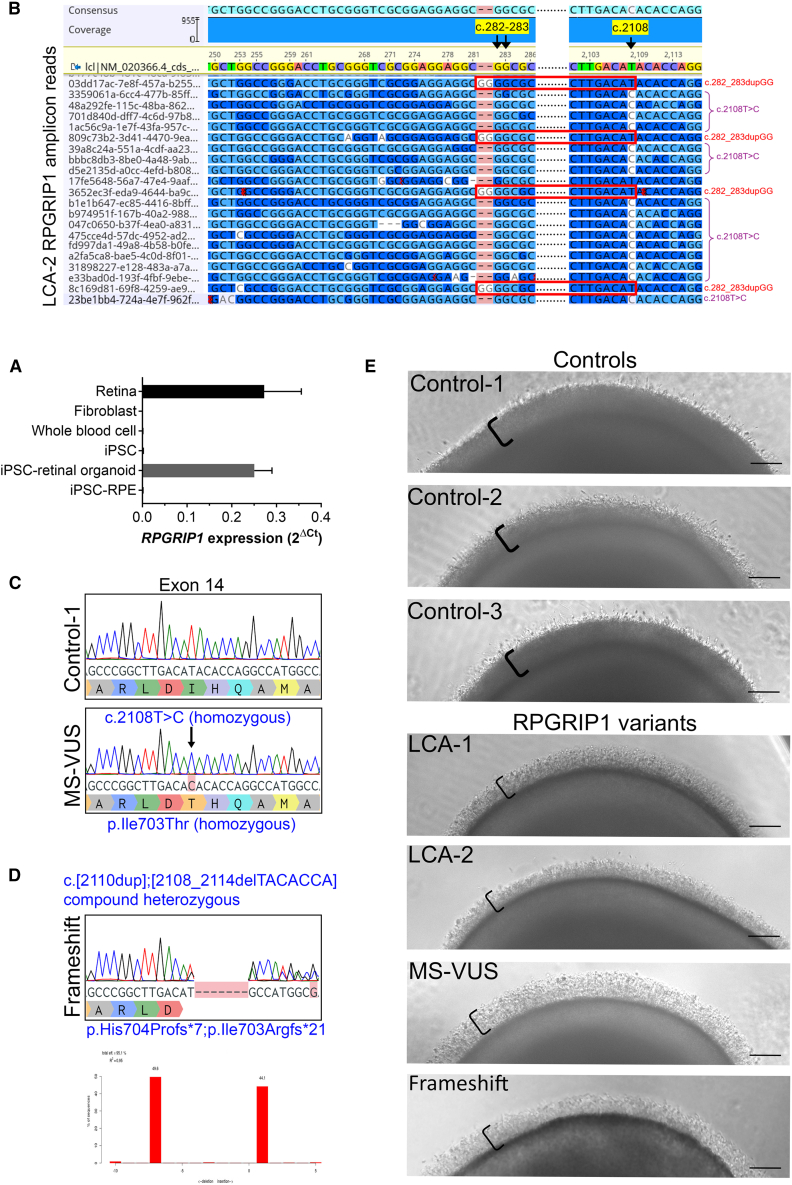
iPSC retinal organoids for RPGRIP1 variant phasing and disease modeling (A) RT-qPCR comparison of relative *RPGRIP1* transcript levels across different control cell types. RPE, retinal pigment epithelium. (B) ONT long-read sequencing of RT-PCR full-length RPGRIP1 amplicons from day-170 LCA-2 retinal organoids. Subset of reads from the 2 affected regions is shown. Reads boxed in red contain the c.282-283dupGG variant but wild-type at position c.2108 (T) while the other reads have the c.2108T>C change. Control organoids were wild type at both positions (Figure S2A). (C) Representative Sanger sequencing traces confirming CRISPR-Cas9 HDR-mediated introduction of the *RPGRIP1* c.2108T>C p.(Ile703Thr) variant homozygously into Control-1 iPSCs for 2 MS-VUS clonal lines. (D) Sanger sequencing of the frameshift clonal sub-line with RPGRIP1 compound heterozygous frameshift variants: −7 deletion and +1 insertion per allele (TIDE analysis, lower). (E) Bright-field images of day-210 retinal organoids. Reduced density of photoreceptor brush borders (square brackets) in all 4 forms of *RPGRIP1* variant lines compared to 3 control lines. Scale bars: 100 μm.

Given the exclusive expression of RPGRIP1 in photoreceptor cells relative to other human somatic cell types (Figure 2A), we used retinal organoids differentiated from iPSCs to study the functional impact of *RPGRIP1* variants. We created patient-derived iPSC lines from patients LCA-1 and LCA-2, focusing on 2 clonal lines per derivation, all of which retained control karyotype, expressed pluripotency markers OCT4, NANOG, and SOX2, and were capable of trilineage differentiation (Figures S1C–S1E).

A well-established 2D-3D protocol (West et al., 2022) was used to differentiate both LCA-1 and LCA-2 lines along with 3 control iPSC lines to retinal organoids. Prior to further characterization, we evaluated LCA-2 retinal organoids for variant phasing of the 2 heterozygous *RPGRIP1* variants. Full-length *RPGRIP1* amplicons (3.8 kb) amplified by reverse-transcription PCR (RT-PCR) from LCA-2 organoid cDNA were sequenced by Oxford Nanopore Technology (ONT) and aligned to *RPGRIP1* reference sequence NM_020366.4 (Figure 2B). Of a total of 1,500 long-reads aligned, the majority were identified to carry either the c.282-283dupGG or c.2108T>C variant but not both, thus demonstrating that the variants occurred in *trans* in LCA-2 and indicating the value of determining the pathogenicity of the MS-VUS.

To examine the effect of the *RPGRIP1*: c.2108T>C p.(Ile703Thr) MS-VUS, we utilized CRISPR-Cas9 homology directed repair to engineer this variant in homozygous form into the Control-1 iPSC line (Figure 2C), yielding 2 clonal sublines. We also examined an additional CRISPR-Cas9-edited clone, the “Frameshift” line, which carried RPGRIP1 compound heterozygous frameshift variants predicted to disrupt protein expression (Figure 2D). All engineered iPSC sub-lines had no detectable off-target effect in ten predicted off-target sites (Table S1; Figure S2B) and developed retinal organoids normally.

At day 210 of culture, all organoids analyzed showed a typical brush border region, which delineates the photoreceptors’ CC and OS (Figure 2E). However, under bright-field microscopy, these brush borders appeared consistently denser in the controls compared to all *RPGRIP1* variant lines from ≥3 independent differentiations (Figures 2E and S3).

### RPGRIP1 abnormality disturbs protein interactions at the CC

**Figure 3:**
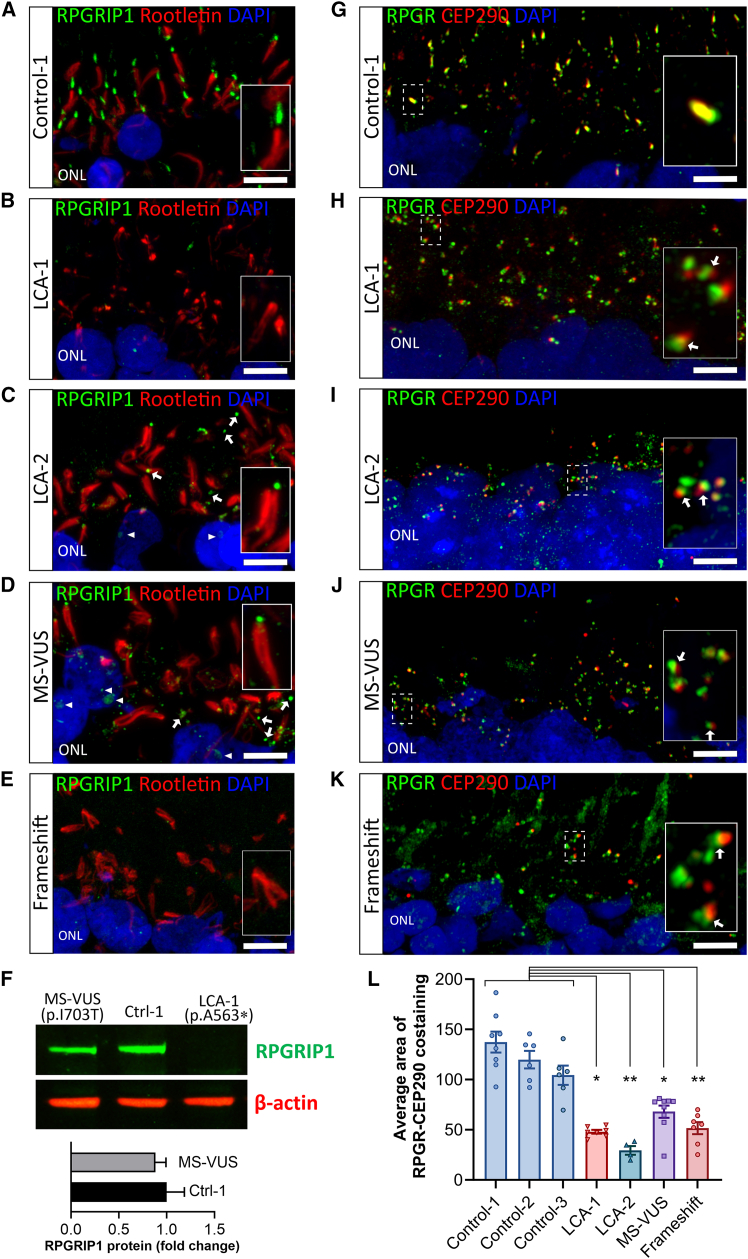
Abnormal RPGRIP1 expression and mislocalization in variant retinal organoids affecting CC proteins (A−E) 63x Airyscan images of unfixed organoid sections immunostained with RPGRIP1 (green) and rootletin (red) from Control-1 (A), LCA-1 (B), LCA-2 (C), MS-VUS (D), and frameshift (E). Inset: zoomed-in images. Abnormal RPGRIP1 staining in (C) and (D): arrows point to examples of mislocalized staining; arrowheads denote staining in photoreceptor nucleoli. (F) Western blot analysis of RPGRIP1 expression (∼160 kDa isoform), 2–3 organoids pooled per lane. Loading control: β-actin. Plot: quantitation of RPGRIP1 protein band intensities normalized to β-actin, versus Control-1. 3 independent blots performed, mean ± SEM. (G−K) RPGR (green) and CEP290 (red) immunostained organoids derived from Control-1 (G), LCA-1 (H), LCA-2 (I), MS-VUS (J), and frameshift (K). Inset: Dashed rectangular regions magnified 600×. Arrows point to where yellow co-staining should be seen in all variant organoids. (L) Quantification of size (pixels) of CEP290 and RPGR yellow co-staining. Organoids from 3 independent differentiation batches: 4–9 retinal organoids per group. Plot show mean ± SEM. ^∗^*p* < 0.05, ^∗∗^*p* < 0.01. Scale bars: 5 μm. ONL, outer nuclear layer.

We assessed the expression of RPGRIP1 protein in day-210 retinal organoids by focusing on the CC of photoreceptors using immunohistochemistry (IHC) and super-resolution (Zeiss Airyscan) confocal fluorescence microscopy. In control organoids, RPGRIP1 was strongly expressed and localized to the CC distal to rootletin, which marks the rootlet of photoreceptors in the ISs, (Figure 3A) but appeared abnormal in all *RPGRIP1* variant lines (Figures 3B–3E). Expression was significantly diminished in LCA-1, as expected due to the homozygous RPGRIP1 nonsense variant (Figure 3B), and in the frameshift organoids (Figure 3E). For LCA-2, RPGRIP1 at the CC was minimal and appeared mislocalized (Figure 3C, arrows), including to the nucleolus of some outer nuclear layer (ONL) nuclei (Figures 3C, arrowheads, S4A, and S4B). These changes were likely derived from the heterozygous *RPGRIP1* c.2108T>C MS-VUS allele of LCA-2 since they appeared more prominent in the MS-VUS organoids homozygous for the variant (Figures 3D and S4A–S4D, *p* < 0.05). Using western blotting, we verified the absence of RPGRIP1 protein in LCA-1 organoids, while levels in MS-VUS organoids were similar to Control-1 (Figure 3F, *p* = 0.58), indicating that the c.2108 T>C change disrupted the correct subcellular localization of RPGRIP1 to the CC rather than affecting overall protein expression.

Since RPGRIP1 functions as an adaptor linking RPGR with CEP290 to form part of the CC interactome (Gerner et al., 2010), we examined the localization of RPGR and CEP290. In all organoid lines, both proteins were correctly localized to the photoreceptor CC (Figures 3G–3K). In all controls, RPGR and CEP290 were positioned close to each other, demonstrating co-staining in yellow (Figure 3G). In contrast, for all forms of variant organoids (Figures 3H–3K), RPGR and CEP290 were noticeably separated from each other and lacked yellow co-staining. Pixel area of co-staining was measured to be larger by at least 2-fold in control organoids compared with variant organoids (Figure 3L, *p* < 0.05). Overall, these results suggest disruption to the CC protein complex when RPGRIP1 is reduced or absent at the CC.

### Disrupted transport of opsins to photoreceptor OSs when RPGRIP1 is lost or mislocalized from the CC

**Figure 4:**
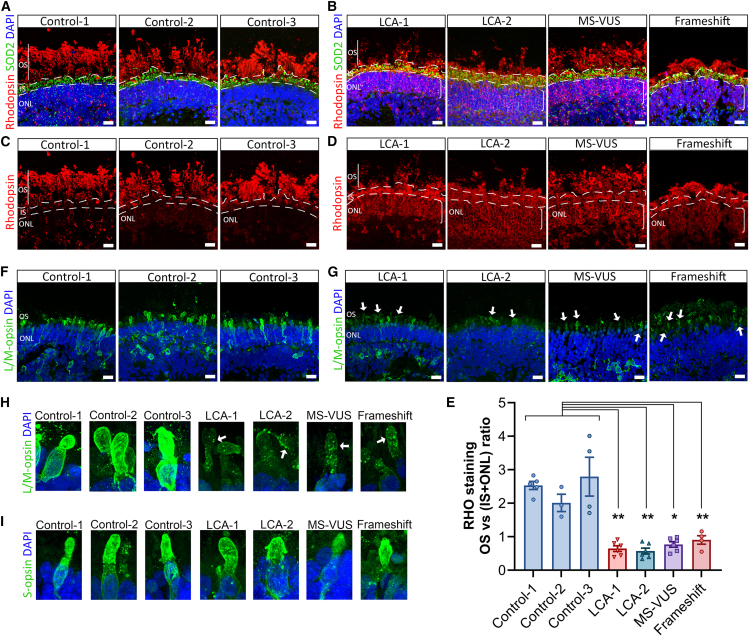
Biomarkers of abnormal photoreceptors in RPGRIP1 variant retinal organoids (A and B) Rhodopsin-immunostained images of organoids from 3 control lines (A) and the 4 RPGRIP1 variant lines (B), which have mislocalized staining in the IS and ONL (square brackets). SOD2 marks the IS of photoreceptors. (C and D) (A) and (B) images shown without SOD2 and DAPI staining. Dashed lines define the beginning of ONL and IS regions. (E) The ratio of rhodopsin in the OS versus the IS and ONL (IS + ONL) of each organoid was calculated from fluorescence intensities measured. Lower ratio values indicate more mislocalized staining in the IS and ONL. Graph shows mean ± SEM. 3 independent organoid differentiation batches: 3–5 organoids per control line; 4–6 organoids per variant line. ^∗^*p* < 0.05, ^∗∗^*p* < 0.01. Scale bars: 20 μm. (F and G) L/M-opsin-immunostained red/green cone photoreceptors of organoids derived from 3 control lines (F) and 4 RPGRIP1 variant lines (G), which have reduced, punctate staining in the OS (arrows). (H) Magnified images of L/M-opsin-positive photoreceptors from (F) to (G). Arrows point to puncta staining. (I) Magnified images of S-opsin-immunostained blue cone photoreceptors of organoids from 3 control lines and 4 RPGRIP1 variant lines. ONL, outer nuclear layer; IS, inner segment; OS, outer segment.

To assess if altered spatial proximity within the CC protein complex could affect transport of cargo from the photoreceptor IS to OS, we analyzed the status of rod and cone opsins in organoids. Rhodopsin was expressed throughout the rod OS of all retinal organoids but appeared fluorescently less intense for LCA-1, LCA-2, and frameshift (Figure 4B) compared with controls (Figure 4A). Furthermore, rhodopsin is clearly seen in the IS of all variant organoids compared to controls (Figures 4A vs. 4B) and is markedly mislocalized to the ONL in all forms of RPGRIP1 variant organoids including the MS-VUS (Figure 4D) compared to controls (Figure 4C). To measure the degree of rhodopsin mislocalization, we compared the ratio of staining in the organoid OS versus the IS and ONL compartments resulting in significantly reduced ratios in all variant organoids relative to controls (Figure 4E), and mislocalized presence in the ONL was also confirmed (Figures S4E and S4F). L/M-opsin also appeared abnormal, indicated by reduced and more punctate staining in the OS of red/green cone photoreceptors from all variant organoids (Figures 4G and 4H, arrows) in contrast to strong and diffuse staining normally observed in control organoids (Figures 4F and 4H). S-opsin-stained blue cone photoreceptors did not show detectable differences between control and variant organoids (Figure 4I). Thus, RPGRIP1 variant organoids, including the MS-VUS line, have disrupted transport of rhodopsin and L/M-opsins from the IS to the OS.

### *RPGRIP1* signature genes associated with poor photoreceptor function and stress response in *RPGRIP1* variant organoids

**Figure 5:**
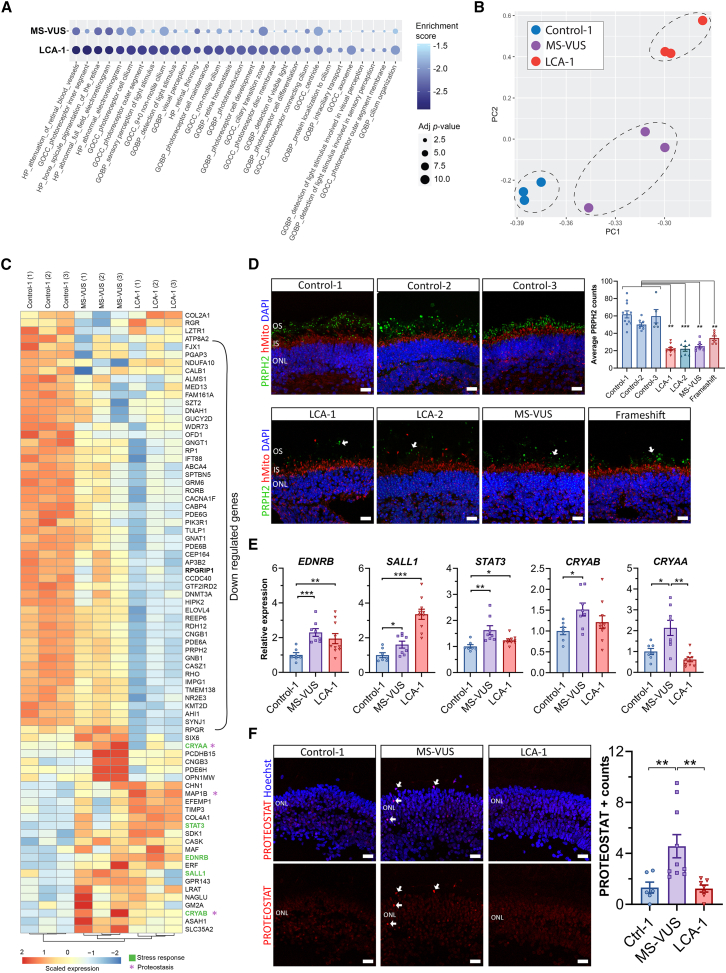
Bulk transcriptomics of week 30 retinal organoids (A) Top-ranking GO terms with GSEA-negative enrichment scores for both LCA-2 and MS-VUS retinal organoids, versus Control-1, are mainly related to retinal biology. Adj *p* value: −Log10 *p* value transformed. (B) Principal-component analysis visualization showing MS-VUS clustering separately from LCA-1 and Control-1 organoids based on the 78 RPGRIP1 signature genes. (C) Unsupervised heatmap of the 78 RPGRIP1 signature genes. Expression levels are row scaled. Each RNA-seq sample derived from RNA pooled from 3 organoids. (D) PRPH2 (green)-immunostained organoids derived from 3 control and 4 RPGRIP1 variant lines. Arrows point to examples of reduced PRPH2 at the OS of photoreceptors distal to the IS marked by mitochondria staining (red). Top right graph: quantification of PRPH2 foci in OS of photoreceptors at 5–8 areas per organoid, *n* = 5–11 organoids per group. (E) RT-qPCR validation of increased expression of stress response genes *ENDRB*, *SALL1*, *STAT3*, *CRYAB*, and *CRYAA* in MS-VUS and or LCA-1 organoids versus Control-1. *n* = 7–10 RNA samples (4–9 pooled organoids) per line. (F) PROTEOSTAT staining (arrows) in the ONL of MS-VUS organoids compared to Control-1 and LCA-1. Graph: quantification of total PROTEOSTAT-positive ONL nuclei per organoid normalized to organoid size. 6–10 organoids per group. 2–3 independent differentiation batches. Plots show mean ± SEM. ^∗^*p* < 0.05, ^∗∗^*p* < 0.01, ^∗∗∗^*p* < 0.001. Scale bars: 20 μm. ONL, outer nuclear layer; OS, outer segment; IS, inner segment.

The morphological biomarkers showing the presence of aberrant photoreceptors in MS-VUS organoids, similar to LCA-1 (pathogenic variant) and LCA-2, suggests that the MS-VUS c.2108T>C p.(Ile703Thr) is likely a disease-causing allele. To gain insights into mechanisms associated with RPGRIP1 dysfunction, we interrogated day-210 retinal organoids by transcriptomics, focusing on the LCA-1 and MS-VUS organoids compared with Control-1. Initially, we used gene set enrichment analysis (GSEA) to perform a global evaluation of transcriptional changes. Comparing each RPGRIP1 variant group to Control-1 organoids identified 1,120 and 349 gene ontology (GO) terms enriched in LCA-1 and MS-VUS organoids, respectively (Figure S5A). Common to both were 185 terms mostly affecting general ubiquitous cellular pathways. However, a subset of gene sets suggests that RPGRIP1-LCA may be associated with cytoskeletal regulation, protein breakdown/cleavage, oxidative stress, and others (Figure S5B). Notably, examining the top-ranking 50 negatively enriched terms (i.e., enriched in the Control-1 organoids, adj *p* < 0.05) shared with LCA-1 and MS-VUS organoids identified mainly retinal gene processes such as those associated with the photoreceptor disc membrane, IS and OS, cilium, and phototransduction (Figures 5A and S5C). This underrepresentation of retinal gene sets corroborates an abnormal phenotype in the LCA-1 and MS-VUS lines. Interestingly, most enrichment scores were more negative with lower adj *p* values for LCA-1 than MS-VUS (Figure 5A), suggesting a hypomorphic effect of the MS-VUS variant.

To identify a transcriptomic signature for RPGRIP1 function, we used a systematic approach to define a set of genes that can discriminate abnormality in RPGRIP1 function using GO terms containing the *RPGRIP1* gene and our transcriptomics data. We identified the overlap of genes between all genes of RPGRIP1-associated GO terms and differentially expressed genes between MS-VUS and Control-1 (*p* < 0.05). The final signature gene set resulted in a total of 78 genes, which led to distinct clustering of the sample types when visualized by principal-component analysis (Figure 5B). Among the 78 signature genes, we saw a striking downregulation of 49 retinal-related genes in LCA-1 and MS-VUS compared to Control-1 (Figure 5C), which is in line with our earlier GSEA results. The extent of downregulation was often larger and more statistically significant in LCA-1 than MS-VUS, suggesting that the former has more severe disease. These include *RHO*, which was downregulated by 71% in LCA-1 and 34% in MS-VUS, respectively, and appears consistent with the lower density rhodopsin staining seen in the OS of LCA-1 and MS-VUS organoids compared to controls (Figures 4A–4D). Peripherin 2 (*PRPH2*) encodes a tetraspanin protein normally expressed in the OS of rod and cone photoreceptors and was also reduced by 53% and 18% in LCA-1 and MS-VUS organoids, respectively. Corroborating this, we identified a 36%–60% reduction of PRPH2 IHC staining in the photoreceptor OS of all RPGRIP1 variant organoids relative to controls (Figure 5D, *p* < 0.05). Intriguingly, *RPGR* was upregulated by 59% (1.6-fold) in the MS-VUS organoids, which was not observed by IHC analysis and may relate to possible disparity that can occur in transcriptomic and protein expression due to factors such as differences in synthesis and degradation and the impact of post-translational modifications. The remaining 26 signature genes were either upregulated in both forms of *RPGRIP1* variant organoids or just the MS-VUS (Figure 5C). Most of these genes could be grouped into categories associated with stress response/prosurvival, proteostasis/cellular metabolism, and protein scaffolding/transport. Evaluating an independent set of organoid samples by quantitative reverse-transcription PCR (RT-qPCR), we validated the increased expression of known stress response genes *ENDRB*, *SALL1*, and *STAT3* in both the LCA-1 and MS-VUS organoids versus Control-1, and *CRYAA* and *CRYAB* transcripts in the MS-VUS organoids only (Figure 5E, *p* < 0.05).

### *RPGRIP1* MS-VUS associated with protein misfolding and increased proteostasis response

Interestingly, both *CRYAA* and *CRYAB* transcripts were highest in the MS-VUS organoids compared with Control-1 and LCA-1 organoids. Crystallins are heat shock proteins, so they also function as molecular chaperones that bind misfolded proteins to prevent aggregation (Horwitz, 2000) suggesting the presence of misfolded RPGRIP1 protein in the MS-VUS organoids. Abnormal levels of misfolded protein aggregations are seen in mouse models of retinitis pigmentosa carrying MS variants of rhodopsin, identified by positive staining with the PROTEOSTAT protein aggregation dye, which fluoresces when trapped in aggregated protein formations (Vasudevan et al., 2024). Using the dye, we detected a 3- to 4-fold higher number of nuclear PROTEOSTAT staining within the ONL of MS-VUS organoids than in Control-1 and LCA-1 (Figure 5F, arrows, *p* < 0.05), thus identifying a novel biomarker associated with protein misfolding due to the *RPGRIP1*: c.2108T>C p.(Ile703Thr) variant.

### Aberrant proportions of photoreceptor subpopulations associated with stress and induced proteostasis in *RPGRIP1* variant organoids

**Figure 6:**
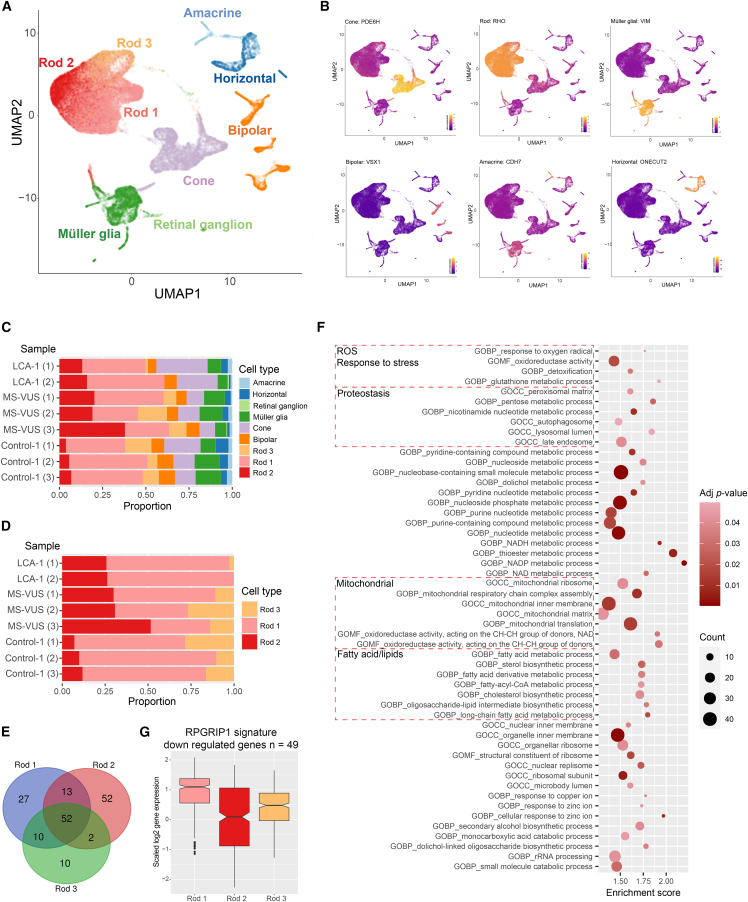
Single-cell transcriptomic comparison of RPGRIP1 variant retinal organoids (A) Unsupervised clustering of single cells and annotation of clusters into cell types of the retina. (B) Examples of key genes that specifically identify the 6 cell types shown. (C) Proportions of retinal cell types present in control and *RPGRIP1* variant retinal organoids. (D) Proportions of the 3 rod subpopulations showing higher proportion of Rod-2 cells in all variant organoids versus controls. (E) Venn diagram representation of the GO terms enriched in Rod-1, 2, and 3 cells by GSEA (*p* < 0.05). (F) Select GSEA-enriched GO terms of interest unique to Rod-2 cells. (G) Mean collective expression of the 49 downregulated RPGRIP1 signature genes was lowest in Rod-2 versus Rod-1 or 3 cells.

To ascertain further molecular mechanisms at the single-cell level, we examined LCA-1, MS-VUS, and Control-1 retinal organoids using single-cell RNA sequencing. After filtering of poor-quality cells, we performed classification of the single cells using a human reference and visualized the results on UMAP (Figure S6A). The identity of these cells was confirmed using known retinal markers and Cepo (Kim et al., 2023) revealing distinct populations of major retinal cell types including rod and cone photoreceptors, amacrine, Müller glial, and horizontal cells (Figures 6A and 6B). Unsupervised clustering of rod photoreceptors showed 3 subclusters of rod photoreceptors. In particular, the composition of the Rod-2 subpopulation was strikingly different between organoids with proportions at least 2-fold higher in both forms of *RPGRIP1* variant organoids compared to Control-1 (Figures 6C and 6D).

To identify the biological significance of these different rod subpopulations, we applied GSEA on all genes ranked by Cepo-derived gene statistics scores for each rod type. We identified 52 enriched GO terms common to all 3 rod subtypes, with 27, 52, and 10 terms unique to Rod-1, 2, and 3, respectively (Figure 6E). Interestingly, the Rod-2 cells, which were proportionally higher in the *RPGRIP1* variant organoids than Control-1, were enriched in GO terms that could be categorized into 4 groups: response to stress, proteostasis, mitochondrial changes, and fatty acid/lipid metabolism (Figure 6F). Response to stress appeared driven by processes relating to oxidative stress, such as metabolism of the antioxidant glutathione, a protective mechanism against oxidative stressed conditions, and oxidoreductase activity, a known major source of reactive oxygen species (Kurutas, 2016). Oxidative stress can disrupt cellular proteostasis, which may also be linked to the removal of unwanted/misfolded protein for degradation via peroxisomes, autophagosomes, and lysosomes important for the maintenance of a healthy protein population within a cell. Rod-1 and Rod-3 cells had similar proportions in variant and control organoids, with Rod-1 cells represented by mostly generic terms not specific to the retina (Figure S6B), and Rod-3 cells appeared to be undergoing replication changes (Figure S6C). Of the 3 rod subpopulations, the scaled mean expression level of 49 downregulated *RPGRIP1* signature genes was lowest in Rod-2 cells (Figure 6G), and combined with their greater presence in disease organoids, suggests that our bulk RNA sequencing (RNA-seq) signature associated with *RPGRIP1* disease may be derived at least in part from Rod-2 cells.

### Improvement of phenotypic biomarkers following AAV-mediated augmentation of RPGRIP1 expression in LCA-1 retinal organoids

**Figure 7:**
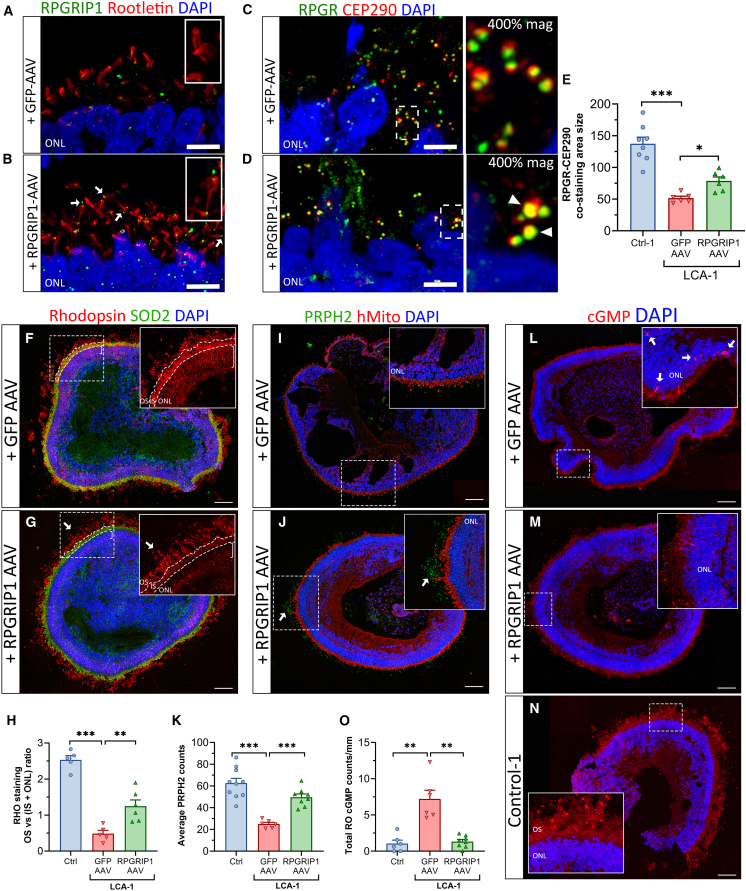
*RPGRIP1*-AAV-mediated gene replacement therapy in LCA-1 retinal organoids improves pathogenic phenotype (A and B) RPGRIP1 (green) and rootletin (red)-immunostained images of LCA-1 organoids 63 days post transduction with GFP-AAV (A) or RPGRIP1-AAV (B). Arrows denote RPGRIP1 staining at the CC distal to rootletin. (C and D) RPGR (green) and CEP290 (red)-immunostained images of LCA-1 organoids transduced with GFP-AAV (C) or RPGRIP1-AAV (D). Arrowheads denote areas of improved yellow co-staining. (E) Average area size (pixel) of RPGR-CEP290 co-staining measured from 4 to 6 63x Airyscan images per organoid. (F and G) Whole-organoid rhodopsin- and SOD2 (IS marker)-immunostained images of LCA-1 organoids transduced with GFP-AAV (F) or RPGRIP1-AAV (G). Inset, enlarged images display rhodopsin only. Improved staining at the photoreceptor OS (arrows) and reduced mislocalization to the ONL (square brackets) due to RPGRIP1-AAV treatment. (H) Quantification of rhodopsin staining intensities in the OS versus the IS and ONL compartments calculated as a ratio per organoid. (I and J) PRPH2- and human mitochondria (IS marker)-immunostained images of LCA-1 organoids transduced with GFP-AAV (I) or RPGRIP1-AAV (J). Arrows indicate increased PRPH2 foci at the photoreceptor OS. (K) Quantification of OS PRPH2 foci within defined set sized area. Mean counts from 5 to 8 areas per organoid. (L–N) cGMP-immunostained whole-organoid images of LCA-1 after GFP-AAV (L) or RPGRIP1-AAV treatment (M) and non-transduced Control-1 organoids (N). Inset: enlarged region shown with arrows pointing to examples of abnormal cGMP staining in photoreceptor somas in LCA-1 organoids treated with GFP-AAV. (O) Total number of cGMP soma staining counted and normalized to size of organoids (mm). *n* = 6–11 retinal organoids per group from 2 independent transductions. Plots show mean ± SEM. ^∗^*p* < 0.05, ^∗∗^*p* < 0.01. Scale bars: 5 μm (C–F), 100 μm (F, G, I, J, and L–N). OS, outer segment; IS, inner segment; ONL, outer nuclear layer.

We next examined whether the biomarkers identified in this study may reflect improvement following RPGRIP1 gene augmentation in LCA-1 RPGRIP1 null retinal organoids. We created an AAV2 expression construct containing human *RPGRIP1* cDNA controlled by the *GRK1* promoter for exclusive expression in photoreceptors (Figure S7A). For comparison, a second construct with eGFP in place of RPGRIP1 was used as an AAV-treated control. Transduced LCA-1 organoids were collected at around day 210 when GFP staining was observed specifically in the photoreceptor layer of cells of retinal organoids that received the GFP-AAV with an estimated transduction efficiency of 47% of photoreceptors (Figure S7B, 47% ± 9.967% GFP-positive cells; *n* = 10 sections, *n* = 4 GFP-AAV organoids). Following treatment, RPGRIP1 localized correctly to the CC of photoreceptors of LCA-1 organoids transduced with RPGRIP1-AAV (Figure 7B, arrow) versus GFP-AAV (Figure 7A). Areas of RPGR and CEP290 yellow co-staining were larger in the CC of photoreceptors from LCA-1 organoids treated with RPGRIP1-AAV (Figures 7D and 7E, arrowheads; *p* < 0.05) than GFP-AAV (Figure 7C). Furthermore, mislocalized rhodopsin in the ONL and IS was significantly reduced (Figure 7G, square brackets) in addition to improved staining in the OS (arrows) of RPGRIP1-AAV-treated LCA-1 organoids compared with GFP-AAV-treated counterparts (Figure 7F). Quantification of rhodopsin staining intensities resulted in an increase in the average OS/(IS + ONL) ratios, with each treated organoid approaching levels similar to those observed in control organoids (Figures 7H and S7D). Restoring RPGRIP1 expression in LCA-1 organoids also led to regions of improved PRPH2 staining at the OS of photoreceptors (Figure 7J, arrows) compared to GFP-transduced controls (Figure 7I). The number of PRPH2 foci counted was nearly doubled (Figure 7K, *p* < 0.05), thus also suggesting partial rescue of phenotype of LCA-1 after RPGRIP1-AAV treatment. Cyclic guanosine monophosphate (cGMP), found mainly in the OS of photoreceptors, is pivotal for phototransduction. In IRD mouse models of *Rho* loss-of-function pathogenic variants, the abnormal accumulation of cGMP in the soma of photoreceptor cells has been linked to photoreceptor degeneration (Arango-Gonzalez et al., 2014). We analyzed whole organoid sections for cGMP and identified intense staining in the somas of photoreceptor cells of untreated LCA-1 organoids (Figure S7C) and those transduced with GFP-AAV (Figure 7L, arrows). Following RPGRIP1-AAV treatment, the frequency of cGMP accumulated in photoreceptor cell soma was reduced (Figure 7M) similar to that observed in unaffected organoids (Figures 7N and 7O, *p* < 0.05), with strong staining at the photoreceptor OS as expected. In addition, the expression of stress response genes *EDNRB*, *SALL1*, *STAT3*, and *CRYAB* was generally downregulated in treated organoids, trending toward levels detected in Control-1 organoids (Figure S7E).

## Discussion

Pathogenic homozygous and compound heterozygous variants in *RPGRIP1* lead to a severe form of IRD and a clinical diagnosis of LCA or early-onset severe retinal dystrophy. VUS are frequently detected in *RPGRIP1*-IRDs and hinder clinical genetic diagnosis and management options. Single-nucleotide variants causing MS changes are common among VUS in *RPGRIP1* and other IRD genes and challenging to study as they rarely affect expression and need functional genomic studies for reclassification. Here, we used human iPSC-derived retinal organoids to establish the phenotype and biomarkers relevant to *RPGRIP1* disease and demonstrate their use in pathogenicity investigation of MS-VUS and gene augmentation therapy applications.

We found that a reduction or absence of RPGRIP1 at the photoreceptor CC, due to either null or MS variants, resulted in a disease-like phenotype affecting both rods and cones. The ciliary protein interactome appeared abnormal in all RGPRIP1 variant organoids and was sufficient to perturb the transport of rhodopsin and L/M-opsin to the OS of photoreceptors, a phenotype that has been described in RPGRIP1 null murine models (Zhao et al., 2003) and other retinal disease organoid models (Bocquet et al., 2023; Chahine Karam et al., 2022; West et al., 2022). While trafficking of S-opsin appeared unaffected, which may be due to difficulty in detecting differences, due to the low abundance of S-opsin in humans (Kallman et al., 2020), there appeared to be an overall effect on cargo transport to the OS leading to a negative impact on photoreceptor development. This is consistent with the observed reduced density of photoreceptor cells at the borders of all RGPRIP1 variant organoids. This was also reflected in the bulk transcriptomic data where there was a consistent underrepresentation of processes related to photoreceptor biology and function. These were driven by the lower expression of a collection of photoreceptor genes, which contributed largely to the RPGRIP1 gene signature that delineated *RPGRIP1* variant organoids from control. We also saw signs of stress in both MS-VUS and LCA-1 organoids through the upregulation of genes reported as effectors of stress response to rod photoreceptor degeneration or injury/light damage in various IRD mouse models (Aisa-Marin et al., 2024; Jiang et al., 2014; Koso et al., 2016; Rattner and Nathans, 2005). Single-cell transcriptomics enabled us to identify in RPGRIP1 variant organoids the overrepresentation of a subpopulation of rod cells that had an oxidative stressed phenotype and reduced expression of retinal genes, suggesting that this population may be particularly relevant in leading to the downregulated gene signature from the bulk transcriptomics. Thus, interrogating transcriptome changes highlighted key biological pathways that may inform disease status caused by *RPGRIP1* variants.

AAV-mediated gene augmentation has been used to rescue *Rpgrip1* loss-of-function animal models in preclinical studies for RPGRIP1-IRD (Lheriteau et al., 2014; Pawlyk et al., 2010). Here, we adopted this approach to validate key disease phenotypes and biomarkers identified in this *in vitro* system and found an overall improvement of photoreceptor morphology of LCA-1 null variant organoids treated with *RPGRIP1*-AAV. Despite enhanced levels of RPGRIP1 protein at the CC, the ciliary interactome was only marginally improved, but this was enough to significantly improve the trafficking of rhodopsin to the OS and reduce mislocalization within the ONL, consistent with the murine gene therapy study (Pawlyk et al., 2010). Increased PRPH2 at the OS, together with reduced expression of stress response genes, provides a further means of validating the relevance of our *RPGRIP1* gene signature in discriminating disease from unaffected. Functionally, there may be disruption to phototransduction in LCA-1 organoids as suggested by the abnormal accumulation of cGMP in photoreceptor cell bodies, which was rescued by *RPGRIP1*-AAV treatment. This aberration may be a secondary response due to loss of RPGRIP1 expression disrupting efficient trafficking of proteins such as rhodopsin to the OS, partially resembling rhodopsin knockout murine IRD models, which have elevated levels of cGMP accumulation (Arango-Gonzalez et al., 2014). The collective improvement of these biomarkers toward control levels in human photoreceptors supports their utility in assessment of therapy outcomes and interpretation of VUS.

Resolving VUS is of paramount importance for patients and crucial for access to current and future genetic therapies, which is relevant for *RPGRIP1* where late-stage preclinical work of a gene augmentation therapy is reported (Odylia Therapeutics, https://odylia.org/). Here, we investigated a previously unidentified MS variant *RPGRIP1*: c.2108T>C p.(Ile703Thr) from our patient with LCA (LCA-2), which we have shown is in *trans* with an *RPGRIP1* pathogenic frameshift variant by long-read sequencing. At the time of preparing this manuscript, a recent *RPGRIP1*-LCA cohort study included one LCA patient who was also identified with this *RPGRIP1*: c.2108T>C p.(Ile703Thr) variant and classified as a VUS by the authors (Daich Varela et al., 2024). Furthermore, another VUS affecting the same amino acid residue, *RPGRIP1*: c.2107A>G (p.Ile703Val), was recently submitted to ClinVar (Variation ID: 3435091), bolstering the importance of our study elucidating the pathogenicity of MS variants in this region.

Residing in the C2-1 domain of RPGRIP1, the c.2108T>C p.(Ile703Thr) VUS, in homozygous form (MS-VUS line), enabled the expression of full-length protein but caused partial mislocalization away from the CC. Because the mutant amino acid residue threonine is slightly smaller and much less hydrophobic than the wild-type residue isoleucine, we speculated 2 possible affects. The first may destabilize the RPGRIP1 protein, as reported for other proteins with the same amino acid change (Zhang et al., 2012). The second outcome may disturb hydrophobic interactions of RPGRIP1 impacting membrane trafficking or anchoring to the CC. Misfolded/unstable forms of protein are often sequestered into other cellular compartments for refolding or degradation and may explain why RPGRIP1 was unusually clustered in the nucleolus of both the MS-VUS and LCA-2 organoids. The nucleolus is a known detainer of misfolded proteins under stress conditions associated with proteostasis (Amer-Sarsour and Ashkenazi, 2019), and elevated levels of *CRYAA* transcripts and PROTEOSTAT staining were seen in the MS-VUS organoids rather than LCA-1 (RPGRIP1-null) or the isogenic Control-1. The utility of these unique biomarkers should be examined in other MS-VUS in *RPGRIP1* and other IRD genes.

Whether the c.2108T>C p.(Ile703Thr) variant also disrupts the role of the C2-1 domain is uncertain. Homology to other proteins like protein kinase C and synaptotagmin I suggests that it may be involved in calcium-dependent phospholipid binding and membrane-targeting processes (Nalefski and Falke, 1996). Thus, reduced hydrophobicity due to the MS variant may cause unstable tethering to a membrane domain in the CC, resulting in the partial mislocalization observed in MS-VUS organoids. Additional mutagenesis work and localization studies would be needed to elucidate a membrane-targeting role of this domain.

Ultimately, reduced levels of RPGRIP1 at the CC due to the c.2108T>C MS-VUS contribute to an abnormal photoreceptor phenotype shared with organoids from patient-derived lines LCA-1 and LCA-2 but not the isogenic Control-1. Notably, from the bulk transcriptomics perspective, the retinal-related GSEA enrichment scores and dysregulation of retinal RPGRIP1 signature genes were generally milder/hypomorphic for the MS-VUS organoids than LCA-1, which is consistent with natural history studies. Patients carrying homozygous *RPGRIP1* MS variants exhibited less severe forms of disease compared to patients with loss of expression/frameshift variants (Beryozkin et al., 2021). As such, the potential of transcriptomics, interrogating a multitude of markers simultaneously, to delineate differing degrees of disease at a measurable scale directed by statistics may prove useful to assist with classifying MS-VUS on a larger scale. To facilitate scale-up, we envisage that emerging technologies and robotic platforms will ease labor and time commitments associated with the organoid differentiation process. In addition, high-throughput editing strategies using prime or base editors open opportunities for saturation variant interpretation of many VUS simultaneously (Tachida et al., 2025).

This study provides valuable biomarkers identified in an *in vitro* iPSC-derived retinal organoid system. Modeling the MS-VUS enabled variant phasing for patient LCA-2 and produced evidence of disease to assist with VUS reclassification. *RPGRIP1*-LCA disease biomarkers, as reported here, may assist with other variant interpretation and evaluate existing and new treatment options for *RPGRIP1-*related IRD.

## Methods

### Generation of patient-derived iPSC lines

This study was approved by the Sydney Children’s Hospitals Network Human Research Ethics Committee, HREC/17/SCHN/323. Following informed consent, peripheral blood was collected from 2 female LCA patients, LCA-1 and LCA-2, carrying *RPGRIP1* variants. Reprogramming into iPSCs was performed as previously described (Fernando et al., 2022). All clonal sub-lines were tested for mycoplasma (MycoAlert Mycoplasma Detection Kit, Lonza Biosciences); their identities were verified using the PowerPlex 16HS System (Promega, CellBank Australia, Westmead, Australia) and genotyped to ensure the presence of *RPGRIP1* variant/s using Sanger sequencing (Australian Genome Research Facility, AGRF, Australia).

### Control iPSC lines

Three control iPSC lines were used in this study. Control-1 (HPSI0314i-hoik_1) (female) was purchased from the European Collection of Authenticated Cell Cultures. Control-2 and 3 were created from unaffected male and female subjects, respectively (Chahine Karam et al., 2022; Nash et al., 2021).

### Culture of human iPSCs and differentiation to retinal organoids

All iPSC lines were maintained on Matrigel (Corning) in Gibco Essential 8 (E8) medium (Thermo Fisher Scientific) and passaged weekly (1:6) using ReLeSR with addition of CloneR2 on the first day (STEMCELL Technologies). Cells with passage numbers <30 were differentiated to retinal organoids using an established protocol (West et al., 2022) with minor modification wherein developing vesicles in suspension cultures were transitioned to serum-free medium, ALT90, from day 130 onward instead of day 90.

### Genome editing

Benchling (2022, https://benchling.com) was used to identify the gRNA (5′ AGCCCGGCTTGACATACACC 3′) that creates a double-stranded DNA cut 3 bases from the *RPGRIP1* c.2108 nucleotide in exon 14. Single-stranded oligonucleotides of the gRNA sequence were phosphorylated and annealed together and cloned into the pSpCas9(BB)-2A-Puro (PX459) V2.0 plasmid (Addgene 62988) using BbsI restriction enzyme (NEB). A homology directed repair template (100 pmol), a 120-mer single-stranded oligonucleotide carrying the c.2108T>C variant (Sigma-Aldrich, Woodlands, USA), and a gRNA-cas9 plasmid (1 μg) were delivered into 4 × 10^5^ Control-1 iPSCs by Amaxa-4D nucleofection, program CB-150 (P3 kit, Lonza). After 24 h selection with 1 μg/mL puromycin, surviving iPSCs were maintained for a further 7 days in E8 medium to form clonal colonies, which were picked and expanded individually in 96-well plate wells. A portion of cells from each clone was lysed for PCR amplification and Sanger sequencing (AGRF) of the genomic region to identify clones carrying homozygous forms of c.2108T>C. Integrity of the top 10 predicted gRNA off-target sites was checked in positive clones by PCR and Sanger sequencing. PCR primer sequences are listed in Table S2.

### Statistical analysis

Statistical significance of all experimental data was carried out using unpaired Student’s *t* test comparing each RPGRIP1 variant line with either the average values of all 3 control organoid lines or only Control-1. Two clonal iPSC lines were used for LCA-1, LCA-2, and MS-VUS for retinal organoid differentiation experiments. At least 3 independent batches of organoid differentiations were performed.

## Resource availability

### Lead contact

Requests for further information, resources, and reagents should be directed to the lead contact, Robyn Jamieson (rjamieson@cmri.org.au).

### Materials availability

All unique/stable reagents generated in this study will be made available by the corresponding author with a completed materials transfer agreement. There are restrictions to availability of the LCA-1, LCA-2, and Control-3 iPSC lines due to consent-use limits.

### Data and code availability

Bulk and scRNA-seq data have been deposited at NCBI GEO under accession numbers GSE293982, GSE201356, and GSE293984.

## Acknowledgments

We are grateful to the patients who participated in this study. We thank Madeline Vereker, Taya Michel, and Reeva Nadkar from the Eye Genetics Research Unit for their assistance with tissue culture. We thank Prof Steinbusch and Dr. De Vente (Maastricht University) for kindly providing their cGMP antibody. We acknowledge our CMRI colleagues Professor Ian Alexander, Dr. Sharon Cunningham, and Professor Leszek Lisowski for their AAV backbone construct. This research was funded in part by 10.13039/501100000925NHMRC grant GNT2013451 and 10.13039/501100025520MRFF grant MRF2008912 with support from 10.13039/501100001108Australian Vision Research, Cure Blindness Australia, 10.13039/501100005662Neil and Norma Hill Foundation, and Wilton Ainsworth and Luminesce Alliance – a not-for-profit joint venture between CMRI, the Sydney Children’s Hospitals Network, and the Children’s Cancer Institute and affiliated with the University of Sydney and the University of NSW.

## Author contributions

Overall conceptualization, R.V.J. and T.H.L.; methodology, T.H.L., H.J.K., R.V.J., A.G.C., and P.Y.; investigation, T.H.L., A.C., H.J.K., M.F., B.M.N., and N.A.; resources, J.R.G. and R.V.J.; writing – original draft, T.H.L. and R.V.J.; writing – review and editing, all authors; supervision, R.V.J., A.G.C., and P.Y.; funding acquisition, R.V.J. and A.G.C.

## Declaration of interests

The authors declare no competing interests.

## Supplemental information

Document S1. Figures S1–S7, Tables S1–S4, and supplemental methods

Document S2. Article plus supplemental information
