# Supplementary material for: Connecting cilium, stress response, and proteostasis abnormalities inform variant and therapy assessment in RPGRIP1 retinal organoids
Source: Stem Cell Reports. 2025 Nov 20;20(12):102717. doi: 10.1016/j.stemcr.2025.102717 (PMC12744860; doi:10.1016/j.stemcr.2025.102717)
Supplement: Document S1. Figures S1–S7, Tables S1–S4, and supplemental methods [file mmc1.pdf]

Stem Cell Reports, Volume *20*

Supplemental Information

Connecting cilium, stress response, and proteostasis abnormalities inform variant and therapy assessment in *RPGRIP1* retinal organoids

To Ha Loi, Anson Cheng, Hani Jieun Kim, Milan Fernando, Benjamin M. Nash, Nader Aryamanesh, John R. Grigg, Pengyi Yang, Anai Gonzalez-Cordero, and Robyn V. Jamieson

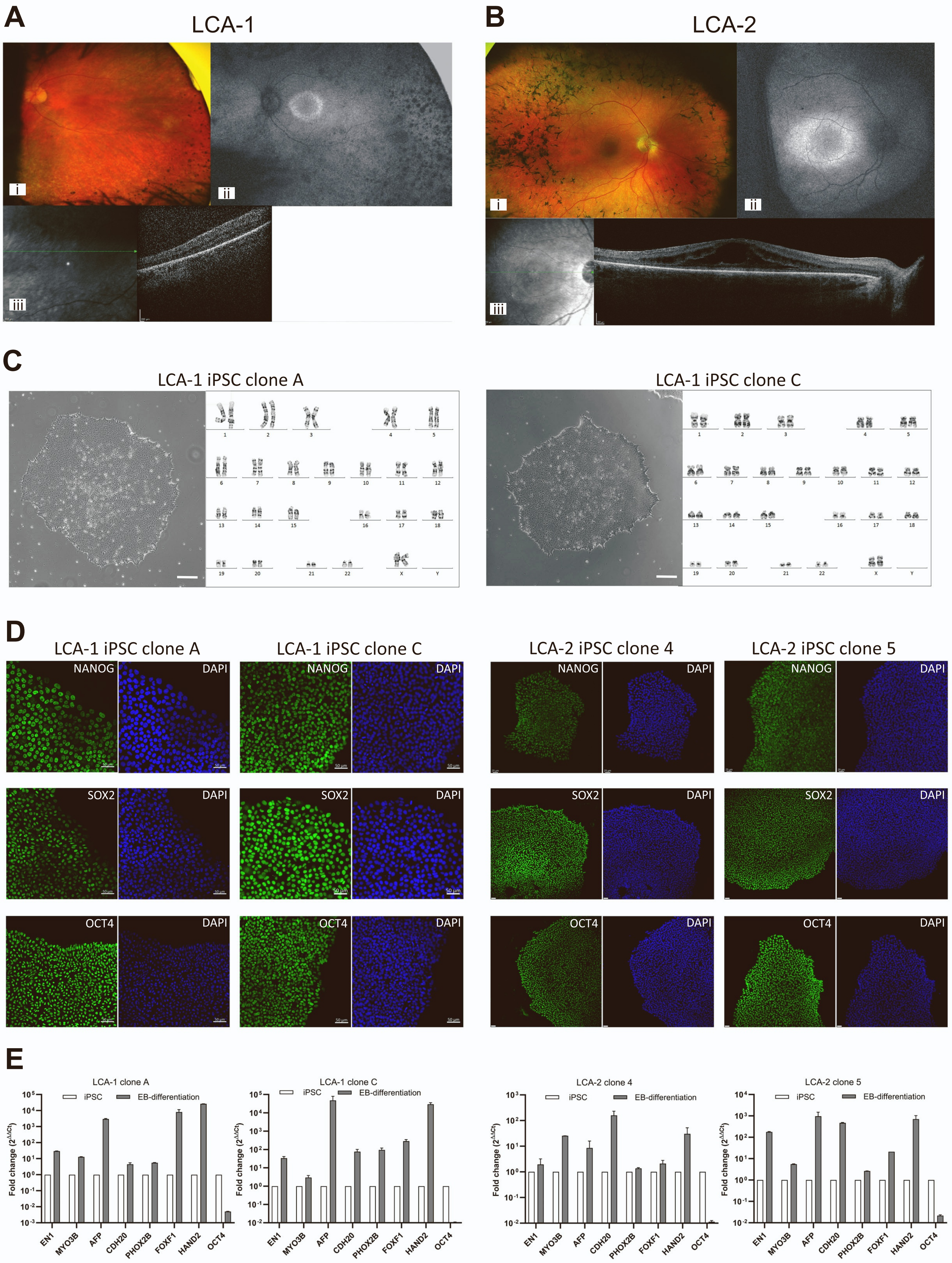

Figure S1. Ophthalmic multimodal imaging of patients LCA-1 and LCA-2 and characterisation of the patient derived iPSC clonal lines.

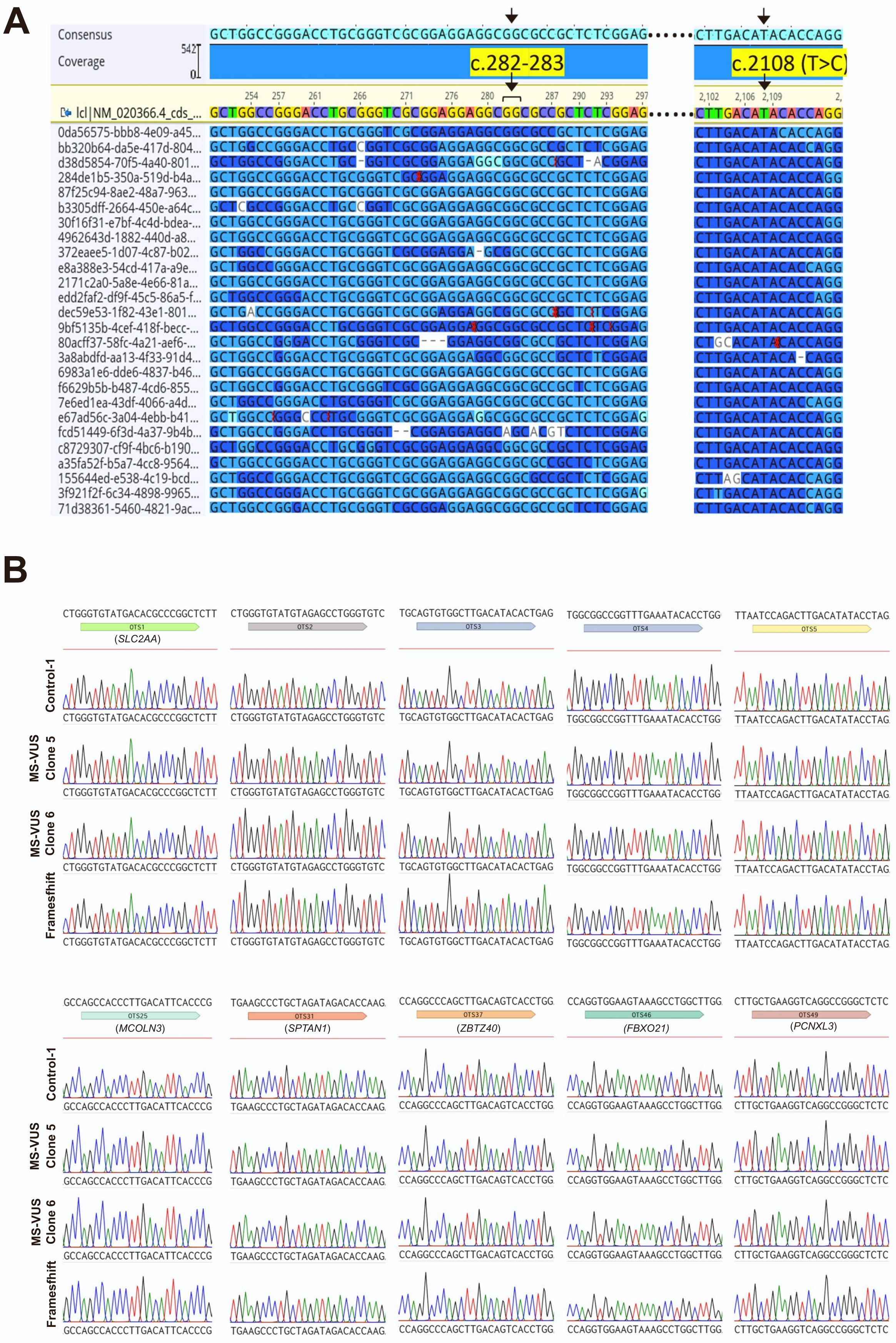

Figure S2. ONT sequencing of normal retinal organoids and assessing off-target sites (OTS) in CRISPR/Cas9 edited iPSC clonal lines.

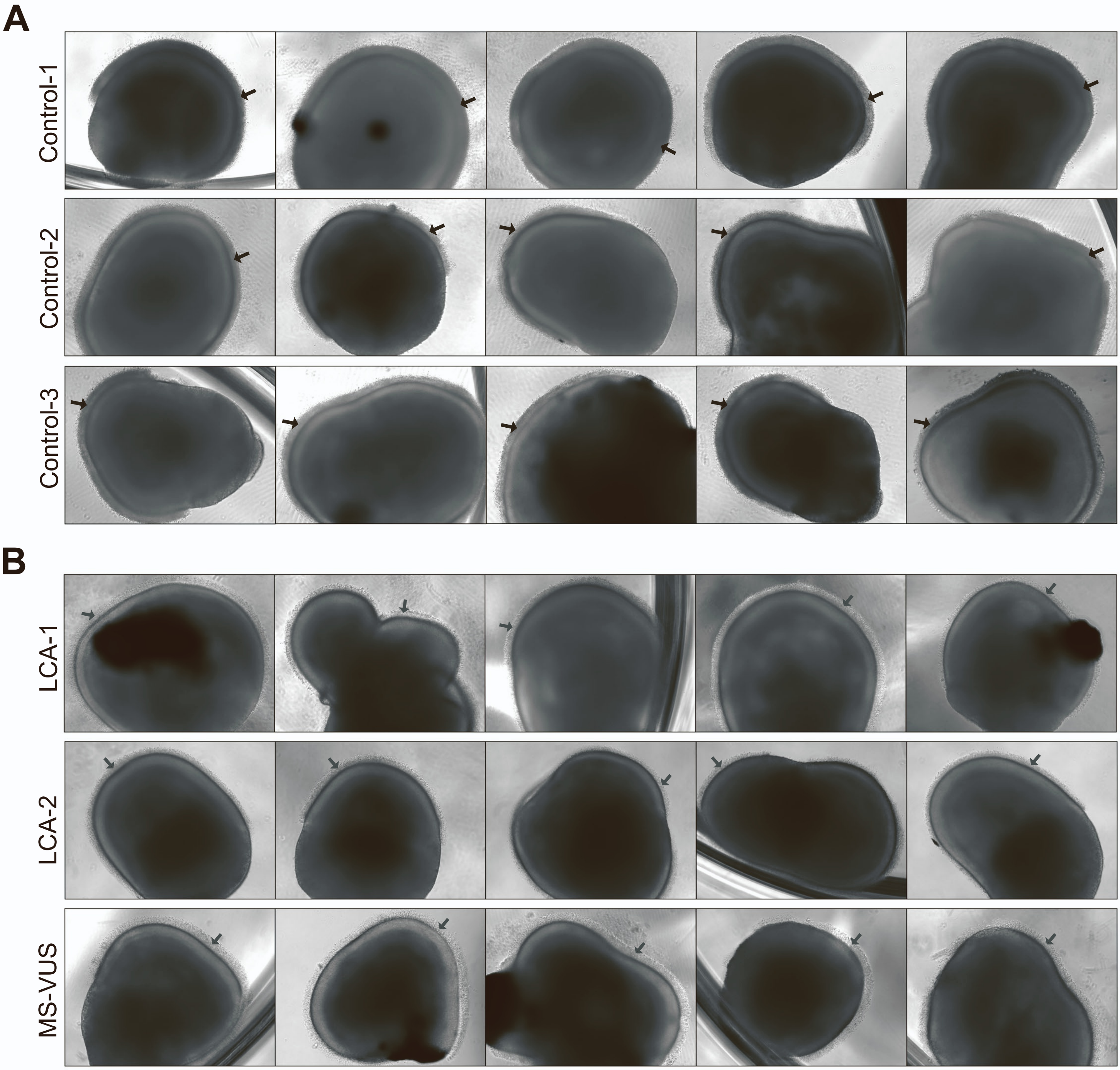

Figure S3. Representative brightfield images of retinal organoids.

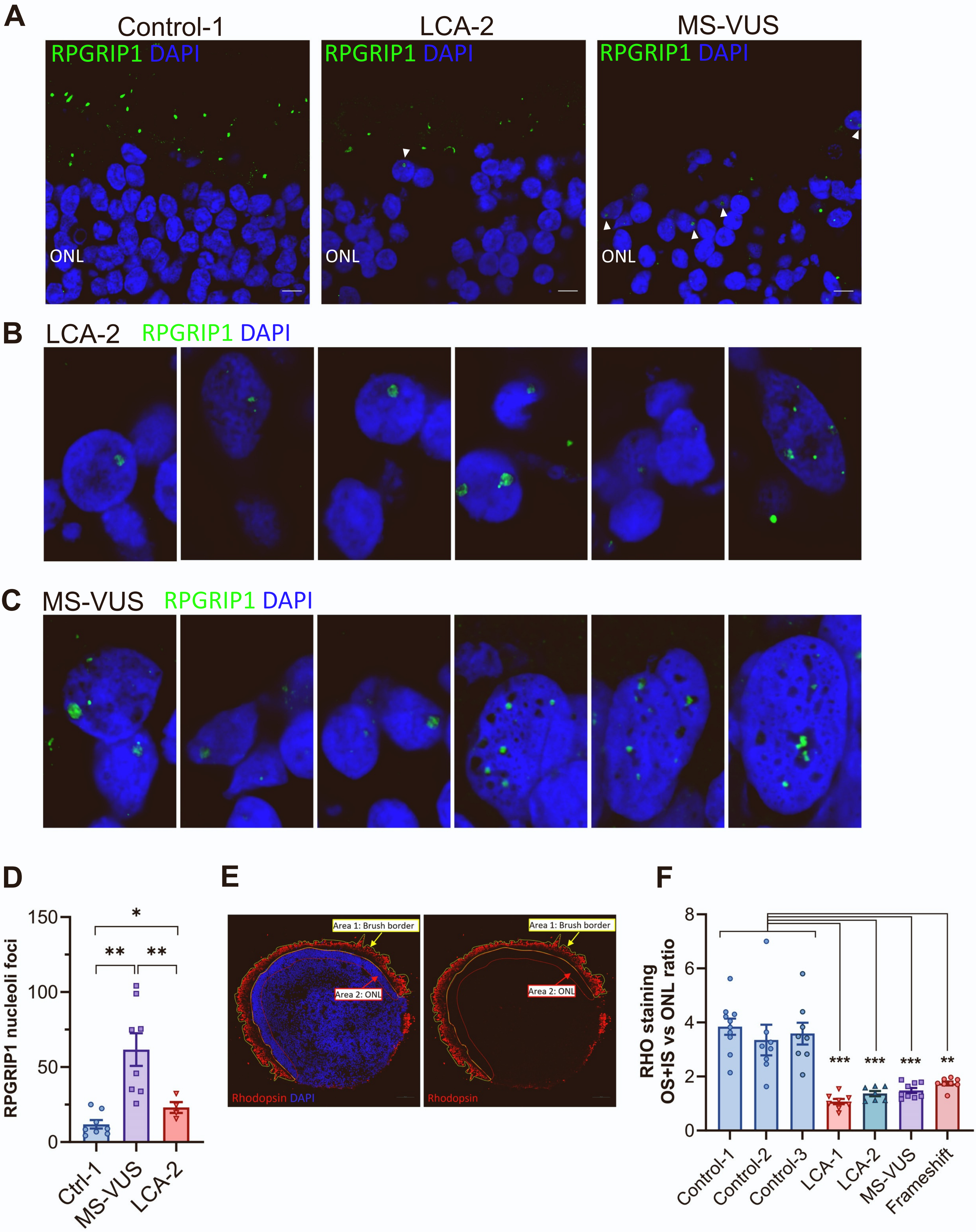

Figure S4. RPGRIP1 in the nucleolus and quantification of RHO staining.

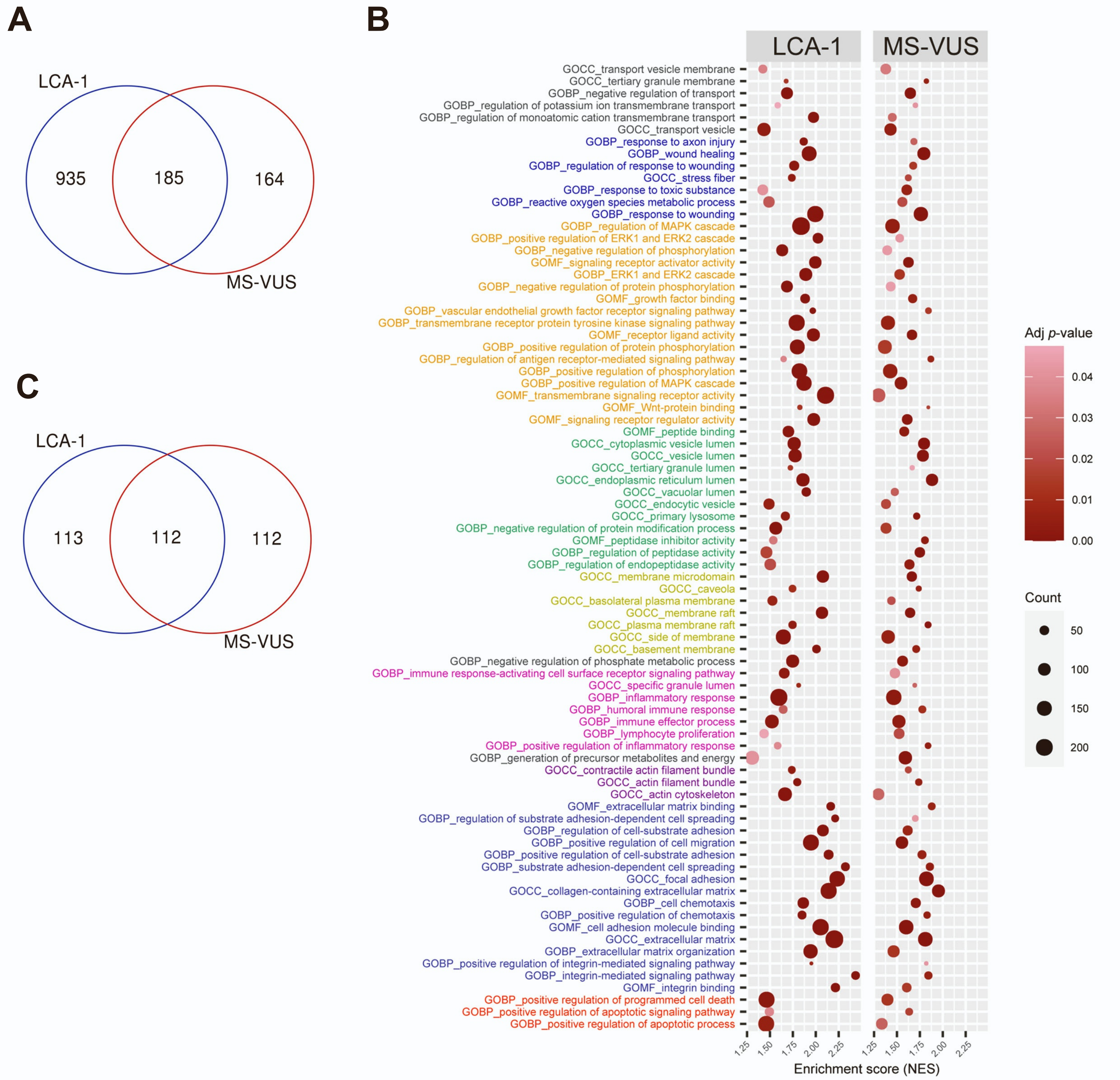

Figure S5. Analysis of bulk RNA sequencing data.

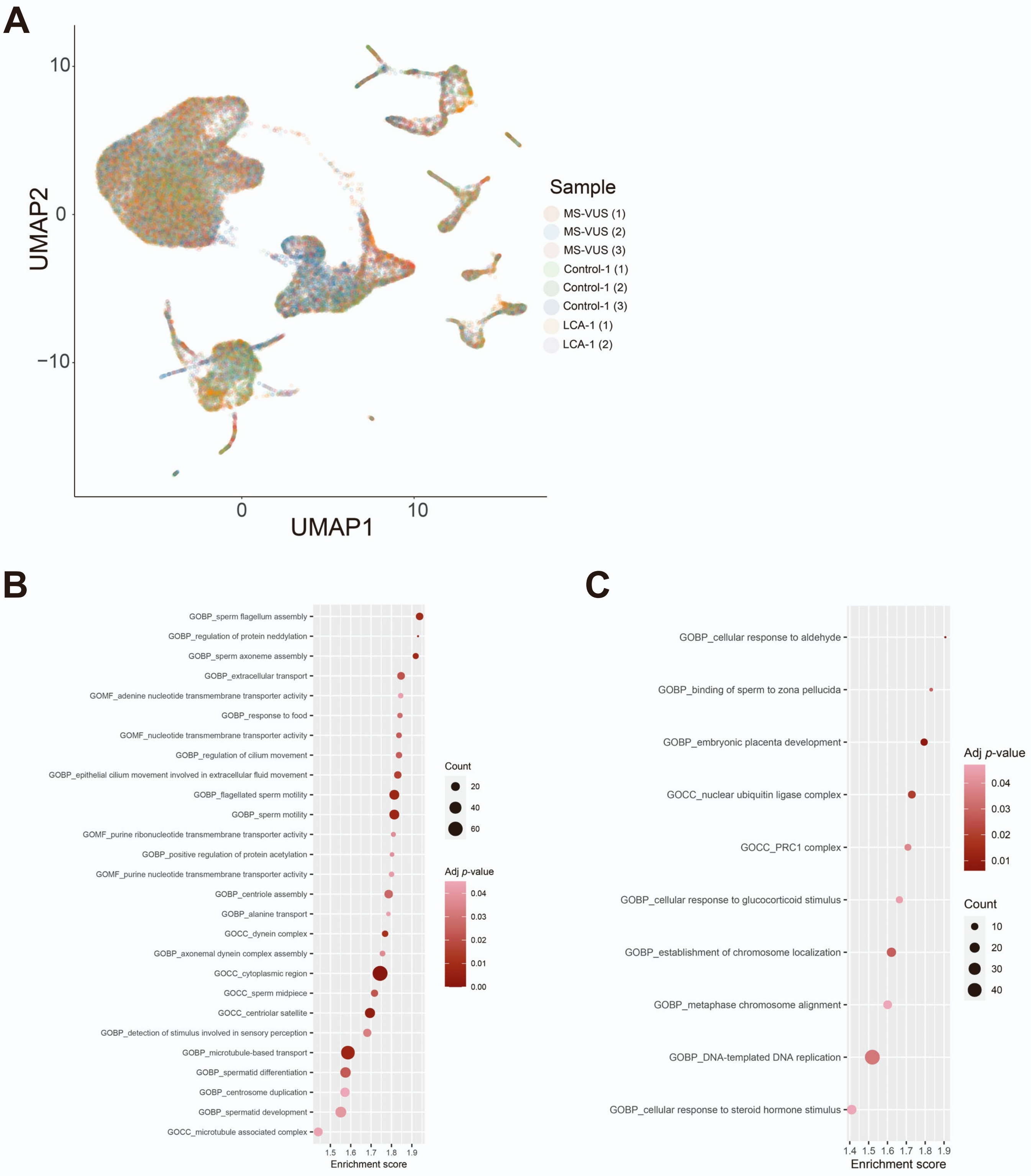

Figure S6. Single cell transcriptomics of RPGRIP1 variant retinal organoids.

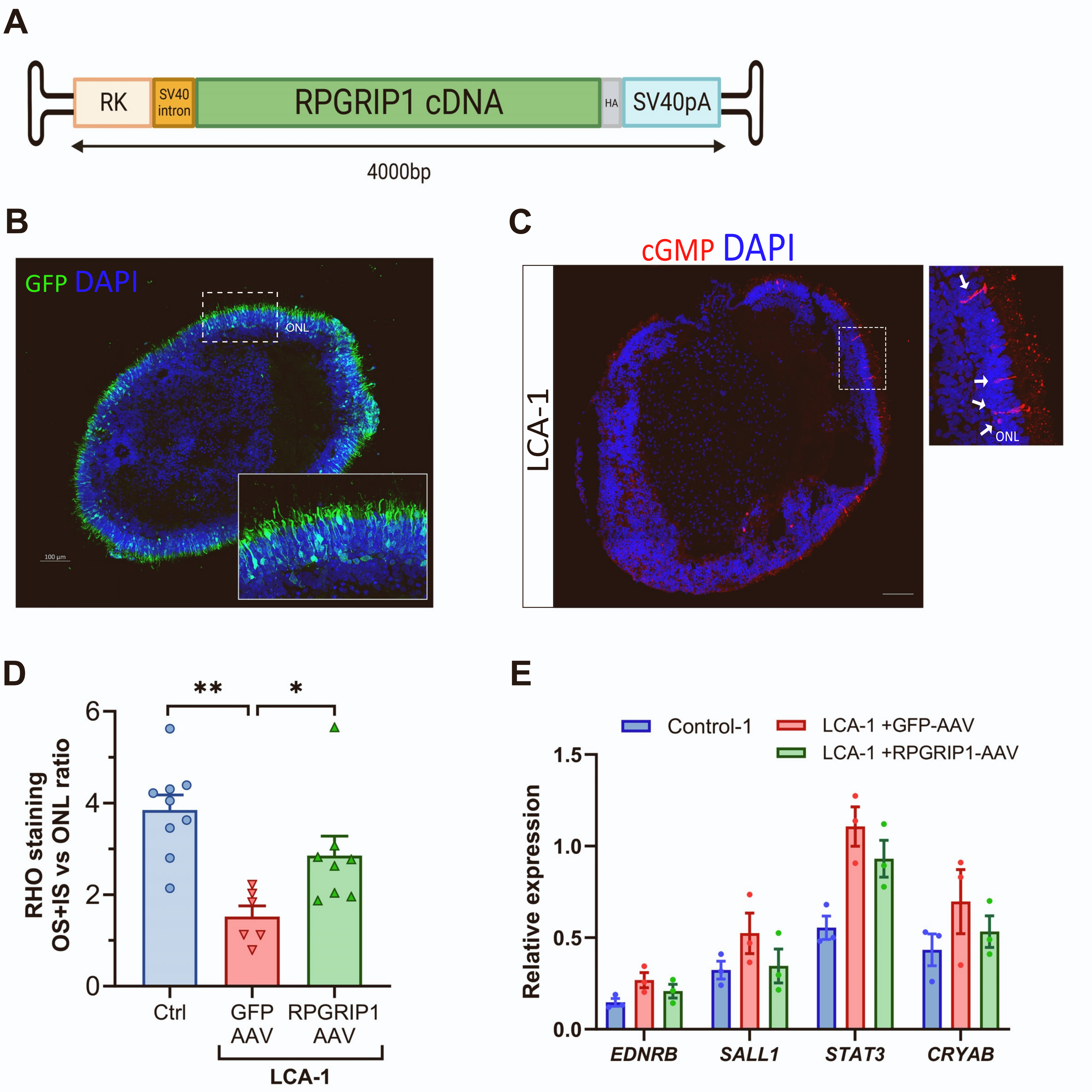

Figure S7. Transduction of LCA-1 retinal organoids with RPGRIP1-AAV and GFP-AAV.

Supplemental Figure Legends

Figure S1. Ophthalmic multimodal imaging of patients LCA-1 and LCA-2 and characterisation of the patient derived iPSC clonal lines.

(A) Patient LCA-1 age 27 years. Imaging acquisition was difficult due to the nystagmus. (i) Ultrawide field pseudocolor image left eye highlighting nummular peripheral retinal pigmentation with patchy diffuse outer retinal atrophy. (ii) Ultrawide field left fundus autofluorescence showing a band of diffuse hyper autofluorescence around the fovea. (iii) Optical coherence tomography (OCT) eccentric scan due to difficulty in fixing. The images show loss of the outer retinal structures. There is no visible ellipsoid zone.
(B) LCA-2 age 29 years. Imaging acquisition was difficult due to the nystagmus. (i) Ultrawide field pseudocolor image right eye highlighting diffuse pigmentary retinopathy with bone-spicule pattern. Significant retinal arteriolar attenuation. (ii) Ultrawide field right fundus autofluorescence showing a broad band of diffuse hyper autofluorescence around the fovea. (iii) OCT right cystoid macular oedema with evidence of outer retina structures with an intact ellipsoid zone.
(C) Typical iPSC colony morphology and control karyotype (46, XX) in all chromosome spreads analysed for LCA-1 iPSC clones A and C. Scale bar = 200µm. Note, the genomic integrity of LCA-2 was determined by digital karyotyping (Victorian Clinical Genetic Services).
(D) Immunofluorescence detection of NANOG, SOX2 and OCT4 pluripotency markers expressed in both clonal iPSC lines for LCA-1 (clones A and C) and LCA-2 (clones 4 and 5). Scale bar = 50µm and 20µm.
(E) Trilineage differentiation capability determined by RT-qPCR showing induced expression of genes expressed in cells from the ectoderm (*EN1* and *MYO3B*), endoderm (*AFP*, *CDH20*, *PHOX2B*) and mesoderm (*FOXF1*, *HAND2*) along with reduced *OCT4* expression in EB-spontaneous differentiation cultures (grey bars) compared to iPSCs (white bars).

Figure S2. ONT sequencing of control retinal organoids and assessing off-target sites (OTS) in CRISPR/Cas9 edited iPSC clonal lines.

(A) Representative ONT long-reads of RPGRIP1 amplicons from Control-1 retinal organoids showing absence of both the c.282_283dupGG and c.2108T>C variants.
(B) PCR amplification and Sanger sequencing of 10 off target sites (OTS) predicted for guide RNA 5' AGCCCGGCTTGACATACACC 3'. Includes the top 5 OTS (OTS-1 to 5) and those affecting genes (*MCOLN3*, *SPTAN1*, *ZBTZ40*, *FBXO21*, *PCNXL3*). Wild type sequences at all 10 sites were retained in both MS-VUS clone 1 and 2 lines and the Frameshift clonal iPSC line compared with Control-1 (unedited parent line). Table S1 lists the top 50 OTS predicted by Benchling.

Figure S3: Representative brightfield images of retinal organoids.

(A – B) Organoid images captured using the EVOS M5000 microscope by 10x objective. Photoreceptor brush borders developing around organoids appeared consistently denser for all 3 control organoids (A) compared with all RPGRIP1 variant lines (B): LCA-1, LCA-2 and MS-VUS. Arrows point to the photoreceptor brush border layer. Organoids from 3 independent batches of differentiations, n = 5 organoids per iPSC line. Control-1 is isogenic to the MS-VUS line. Some organoids have also developed pigmented retinal pigment epithelium (RPE) cells in areas appearing black.

Figure S4: RPGRIP1 in the nucleolus and quantification of RHO staining.

(A) Representative single Z-positioned (non-stacked) 63x airy scan images of RPGRIP1 staining (green) of retinal organoids. Arrowheads point to diffuse/clustered RPGRIP1 staining in the nucleolus of LCA-2 and MS-VUS organoids compared to control-1 (isogenic to the MS-VUS line). Nucleoli are identified by non-DAPI stained black cavities within nuclei. Scale bar: 5µm.
(B – C) Zoomed in images of nuclei from the ONL of (A) and other images are shown for LCA-2 and MS-VUS organoids.
(D) Quantification of RPGRIP1 foci staining in nucleoli of the ONL. Foci count normalised per 100 nuclei counted, plot show mean with ± SEM. 2-3 independent differentiation batches. 4-8 retinal organoids per line. ** $p < 0.01$.
(E) Method of quantifying rhodopsin staining in the photoreceptor ONL versus brush border (OS and IS) of organoids. Representative whole organoid images indicate the 2 areas where rhodopsin (red) fluorescence intensities are measured. Area 1, outlined in yellow, is the brush border of the organoid comprising of photoreceptor IS and OS regions. Area 2, outlined in red, is the DAPI positive ONL corresponding to area 1. Mean integrated intensities from both areas are expressed as a ratio of Area 1: Area2 (Brush border OS+IS:ONL staining). Ratios are calculated within each individual whole-organoid image.
(F) Quantitation of rhodopsin staining intensities in the photoreceptor inner and outer segments (IS+OS) versus the ONL of each organoid. Lower ratio values indicate more mislocalised staining in the ONL. Plot show mean with ± SEM. 3 independent organoid differentiation batches: 7-10 retinal organoids per group. ** $p < 0.01$, *** $p < 0.001$. Scale bar: 20µm. ONL: outer nuclear layer. OS: Outer segment. IS: Inner segment.

Figure S5: Analysis of bulk RNA sequencing data.

(A) GSEA of LCA-1 and MS-VUS versus control-1 organoids. Venn diagram representation of the number of enriched GO terms unique to each variant type or intersecting both types. Adj $p < 0.05$.
(B) Selected enriched GO terms shown (80), including those affecting pathways associated with response to oxidative stress (dark blue), cell signalling pathways (orange font), protein digest/proteolysis (green), lipids/membranes (khaki), immune response (pink), cytoskeletal (purple), adhesion/chemotaxis (blue) and apoptosis (red). Adj $p < 0.05$.
(C) Venn diagram representation of the number of negatively enriched GO terms unique to each variant type or common to both types. Adj $p < 0.05$.

Figure S6: Single cell transcriptomics of RPGRIP1 variant retinal organoids.

(A) UMAP visualisation of single cell clusters coloured by sample type showing uniform representation in all clusters.
(B) Select enriched GO terms unique to Rod 1 (27 terms) and
(C) Rod 3 (10 terms) cell types resulting from GSEA of ranked CEPO statistics. Adj $p < 0.05$.

Figure S7: Transduction of LCA-1 retinal organoids with RPGRIP1-AAV and GFP-AAV.

(A) Schematic of the AAV expression cassette containing RPGRIP1 cDNA driven by the rhodopsin kinase (GRK1) promoter.
(B) GFP staining in the photoreceptor layer of LCA-1 retinal organoids 78 days post transduction with AAV carrying a photoreceptor-specific GRK1-eGFP expression cassette. The AAV-GFP transduction

efficiency was shown by the percentage of anti GFP positive cells in the photoreceptor layer of retinal organoid sections, with this being 47% ± 9.967% (mean ± SD; n = 10 sections, n = 4 organoids). ONL: outer nuclear layer.

(C) Representative whole-organoid image of non-treated day 210 LCA-1 organoids immunostained with cGMP. Inset: enlarged region shown. Arrows pointing to examples of abnormal cGMP staining in photoreceptor somas (total count = 8.45 per mm ± 3.2, mean ± SD).

(D) Quantification of rhodopsin staining in the IS and OS regions versus the ONL calculated as a ratio per organoid. n = 6-11 retinal organoids per group from 2 independent transductions. Plots show mean with ± SEM. * $p < 0.05$, ** $p < 0.01$. IS: inner segment. OS: outer segment. ONL: outer nuclear layer.

(E) RT-dPCR assessment of *ENDRB*, *SALL1*, *STAT3* and *CRYAB* stress response transcripts in Control-1 and AAV transduced-LCA-1 organoids. Expression levels normalised to *HPRT,* mean ± SEM are shown. Organoids: n = 3 per group. Expression levels in RGPRIP1-AAV versus GFP-AAV transduced groups were generally trending towards levels detected in Control-1 organoids although this did not reach statistical significance.

Table S1: The top 49 OTS predicted by Benchling for guide RNA AGCCCGGCTTGACATACACC.

Sequence	PAM	Score	Gene	Chromosome	Strand	Position	Mismatches	On-target
AGCCCGGCTTGACATACACC	AGG	100.00	RPGRIP1	chr14	1	21324966	0	TRUE
AGCCGGGCGTGTCATACACC	CAG	0.83	SLC22A1	chr6	-1	160122313	3	FALSE
ACCCAGGCTCTACATACACC	CAG	0.82		chrX	-1	71992244	4	FALSE
AGTGTGGCTTGACATACACT	GAG	0.81		chr15	1	94743136	4	FALSE
CGGCCGGTTTGAAATACACC	TGG	0.67		chr14	1	104343030	4	FALSE
ATCCAGACTTGACATATACC	TAG	0.49		chr20	1	1637134	4	FALSE
AGCCATGATTAACATACACC	TGG	0.49		chr12	-1	57237710	4	FALSE
AACCTGGATTGACAGACACC	AAG	0.48		chrX	-1	64229965	4	FALSE
AGGCAGTCTTGACATAGACC	AAG	0.47		chr1	1	55911774	4	FALSE
AGCGGGGCTGGACACACACC	TGG	0.42		chr14	1	64733041	4	FALSE
AGACCAGCTAGACATAAACC	AAG	0.39		chr2	1	151541562	4	FALSE
ATCCAGGTTTGACATTCACC	TGG	0.31		chr2	-1	213866616	4	FALSE
AACCAGGCTTGACATACAAT	CAG	0.31		chr2	1	176769652	4	FALSE
AGCCTGGCTCCAGATACACC	AGG	0.30		chr3	1	43374763	4	FALSE
AGCCGGTGTTGACAGACACC	AGG	0.29		chr18	1	65751390	4	FALSE
AGCCATGCCTGACATACACG	AGG	0.29		chr3	-1	4512927	4	FALSE
AGCCAGGCTGGACATACAGA	TGG	0.24		chr4	-1	9832460	4	FALSE
AGCCTGGCTGGACATACAAT	GAG	0.24		chr5	-1	93814256	4	FALSE
AGCCTGTCTTGTCATATACC	TGG	0.23		chr8	1	84047490	4	FALSE
AGCCTGTCTTCACACACACC	AAG	0.17		chr17	-1	45866101	4	FALSE
AGCCCAGCAGGACAAACACC	CAG	0.14		chr16	1	55778209	4	FALSE
AGCCCAGCAGGACAAACACC	CAG	0.14		chr16	1	55734781	4	FALSE
AGCCAGGCCTGGCACACACC	AAG	0.13		chrX	-1	71669257	4	FALSE
AGCCTGGCTTGACGCACACC	GAG	0.12		chr1	-1	14972114	3	FALSE
AGCCGGGCTTCCCAGACACC	TGG	0.12		chr8	-1	141422386	4	FALSE
AGCCACCCTTGACATTCACC	CGG	0.12	MCOLN3	chr1	1	85047433	4	FALSE
AGCCCCACGTGACACACACC	CAG	0.11		chr3	-1	44934121	4	FALSE
AGCCAGGCCTGAAAAACACC	AAG	0.10		chr10	-1	32205796	4	FALSE
AGCCCGGGGTGAAAAACACC	AGG	0.10		chr20	-1	43389449	4	FALSE
TGCCAGGCTTGACAGCCACC	AGG	0.09		chr3	-1	149536602	4	FALSE
AGACCGGCTGGACAGACCCC	GGG	0.09		chr19	1	40796531	4	FALSE
AGCCCTGCTAGATAGACACC	AAG	0.09	SPTAN1	chr9	1	128578232	4	FALSE
AGCACTGCTTGACATATCCC	TGG	0.09		chr8	-1	133786041	4	FALSE
TGCCCGGCTTCTCCTACACC	GAG	0.07		chr12	-1	67478163	4	FALSE
AGCCCAGCTGGACTTACACA	CAG	0.06		chr17	-1	81522766	4	FALSE
AGCCTGGCTTGAGATACTCA	GGG	0.06		chr1	1	29806932	4	FALSE
AGCCCCACTTGACATCCACT	CAG	0.06		chr10	-1	42878318	4	FALSE
GGCCCAGCTTGACAGTCACC	TGG	0.06	ZBTB40	chr1	1	22524377	4	FALSE
AGCCCTGCTGGACACACCCC	TGG	0.05		chr3	1	13048658	4	FALSE
AGAGCGGCTTGACGTGCACC	AAG	0.05		chr14	1	33704774	4	FALSE
AGCTCGCCTTGACTCACACC	GAG	0.05		chr4	1	7202590	4	FALSE
AGCCCGGCTGGACACAGAAC	AAG	0.05		chr20	-1	48266654	4	FALSE
AGCCAGGCTTGACAGAGGCC	AGG	0.04		chr17	-1	79571350	4	FALSE
AGCCCGGCTTAGCATCCACA	CGG	0.03		chr16	1	86198339	4	FALSE
AGCCTGGCTTGGCATCCTCC	GAG	0.03		chr7	1	27105710	4	FALSE
AGCCAGGCCTGACGTCCACC	TGG	0.03		chrX	-1	9751182	4	FALSE
AGCCAGGCTTTACTTCCACC	TGG	0.02	FBXO21	chr12	-1	117165508	4	FALSE
AGCCCAGCTTGGCAGGCACC	AGG	0.02		chr6	-1	46764953	4	FALSE
AGCCCAGCTTGACTCACATC	AAG	0.01		chr2	1	130264496	4	FALSE
AGCCCGGCCTGACCTTCAGC	AAG	0.01	PCNXL3	chr11	-1	65623641	4	FALSE

Table S2: Sequences of primers for genomic DNA PCR.

	Target	Forward primer (5' - 3')	Reverse primer (5' - 3')
Exon 3 variant region	*RPGRIP1*	GTGTACTGGGGACAGAAGGC	AGGCAGAAAGGAGGGAGTGA
Exon 13 variant region	*RPGRIP1*	GACCTAGCCAGTGCCACATT	AGTGGAACACAGGCGTTAGC
Exon 14 variant region (inc CRISPR / cas9 cut site)	*RPGRIP1*	TTTTGAACTGCACATCCACCA	AGATGGTGTGGCAAGGATCAAG
CRISPR/Cas9 predicted off target sites	*OTS1*	GCATCGTCTTCCTGGGTTTCA	TCAGCTTCCTCCTCAAAGACC
	OTS2	GGGCCACATGAGTTGCAGAT	GGGCAGAAACAGGGAGACAA
	OTS3	TCATAGGCTGAACGTAGGGAA	TGACAACCCAAAGAGAGCCTG
	OTS4	CCTGCCCCTTTGACGATGTG	TTGGTGAGTCAATGGGCCT
	OTS5	GCCACCATGAGACCTGCAAC	CTGGCAGAAATGCTAAACTGCAA
	OTS25	AACAAACTAGCCACACACCA	TGCATAACTTTGTCGCCAACTG
	OTS31	TCAGCCTTCTCTTGCTTTCCTT	CGCCAGAGCTTGTCTGTTTG
	OTS37	AATGTGGATCCGCCTCCAAG	TTCTGTTCTGTTTTGTAAGAAGTCC
	OTS46	TGGTTGAGTAGCGGGTACAG	CGTTGAGCTGAGAAGAAATACCAG
	OTS49	GCAATACAATGGTCGCTGGAG	GCTCCACCACTAGGCAGTTA

Table S3: Sequences of primers for RT-qPCR, RT-PCR or RT-dPCR.

	**Target**	**Forward primer (5'-3')**	**Reverse primer (5'-3')**	**Source**
Pluripotency Markers (RT-qPCR)	*NANOG*<br>*OCT4*<br>*SOX2*<br>*MYC*<br>*KLF4*	CCTCCAGCAGATGCAAGAAC<br>AGAAGCTGGAGCAAAACCCG<br>ATGTCCCAGCACTACCAGAG<br>CTGAAGAGGACTTGTTGCGGAAAC<br>GGTCGGACCACCTCGCCTTACAC	AAGGCTGGGGTAGGTAGGTG<br>TCCCAGGGTGATCCTCTTCT<br>GCACCCCTCCCATTTCCC<br>TCTCAAGACTCAGCCAAGGTTGTG<br>CTCAGTTGGGAACTTGACCA	Nash et al.
House-Keeping Genes	*HPRT*<br>*POLR2A*	GACCAGTCAACAGGGGACAT<br>GTGCGGCTGCTTCCATAA	CCTGACCAAGGAAAGCAAAG<br>GCACCACGTCCAATGACAT	Nash et al.
Ectoderm markers	*EN1*<br>*MYO3B*	CGTGGTCAAAACTGACTCGC<br>AAGTCGGTTTCCCCAAGCAA	CGCTTGTCCTCCTTCTCGTT<br>TCAGGACCACAACCACATCG	Nash et al.
Endoderm markers	*AFP*<br>*CDH20*<br>*PHOX2B*	TGAGCACTGTTGCAGAGGAG<br>TGATAACCCACCCCGCTTTC<br>CATCTACACTCGGGAGGAGC	GTTCCAGCGTGGTCAGTTTG<br>AAGGCATCTGCACCATCTCC<br>CCTCTTTGCTCTCGTCGTCC	Nash et al.
Mesoderm markers	*FOXF1*<br>*HAND2*	TGCACCAGAACAGCCACAA<br>CCAGCTACATCGCCTACCTC	TGCTGGTGGTAGTAGGAGCC<br>CCGGCCTTTGGTTTTCTTGT	Nash et al.
Full-length RT-PCR (3861bp)	*RPGRIP1*	ATGTCACATCTGGTGGACCCTA	TCATGAAAACAAATCTTCAGTC	This paper
RT-qPCR (153bp)	*RPGRIP1*	TGAAGGAGTTTCAGGAGAGAGT	GCTGTAGCTGTTCCGCTATG	This paper
Stress response genes	*CRYAA*<br>*CRYAB*<br>*EDNRB*<br>*SALL1*<br>*STAT3*	GGAGATCCACGGAAAGCACA<br>CCGCCTCTTTGACCAGTTCT<br>CCTGCTGCACATCGTCATTG<br>GCCCTGCAGATTCACGAGAG<br>GGTGCCTGTGGGAAGAATCA	GCAGACAGGGAGCAAGAGAG<br>GAACCTGTCCTTCTCCAGGC<br>ACAGTGATTCCCACAGAGGC<br>AACTTGACGGGATTGCCTCC<br>GACATCCTGAAGGTGCTGCT	This paper

Table S4: Antibodies used in the study.

Antibodies	Source	Identifier
Anti-Opsin (Rhodopsin)	Sigma	Cat# O4886,<br>RRID: AB_260838
Anti-Opsin, blue (Rabbit)	Millipore	Cat# AB5407,<br>RRID: AB_177457
Anti-Opsin Antibody, Red/Green (Rabbit)	Millipore	Cat# AB5405,<br>RRID: AB_177456
Anti-RPGRIP1	Thermo Fisher Scientific	Cat# PA5-60172,<br>RRID: AB_2646716
Anti-RPGR (rabbit polyclonal)	Sigma-Aldrich	Cat# HPA001593,<br>RRID: AB_1079835
Anti-CEP290 (B-7)	Santa Cruz Biotechnology	Cat# sc-390462,<br>RRID: AB_2890036
Anti-PRPH2	Thermo Fisher Scientific	Cat# PA5-56154,<br>RRID: AB_2645979
Anti-human Mitochondria, clone 113-1	Millipore	Cat# MAB1273,<br>RRID: AB_94052
Anti-SOD2 (rabbit polyclonal)	Thermo Fisher Scientific	Cat# PA1-31072,<br>RRID: AB_2286296
Anti-cGMP (sheep IgG)	Gift from Prof Steinbusch and Dr De Vente, Maastricht University, NL	N/A
Anti-Rabbit IgG (H+L) Highly Cross-Adsorbed Donkey Secondary Antibody, Alexa Fluor™ 488	Thermo Fisher Scientific	Cat# A-21206,<br>RRID: AB_2535792
Anti-Mouse IgG (H+L) Highly Cross-Adsorbed Donkey Secondary Antibody, Alexa Fluor™ 594	Thermo Fisher Scientific	Cat# A-21203,<br>RRID: AB_2535789
Anti-Sheep IgG (H+L) Cross-Adsorbed Donkey Secondary Antibody, Alexa Fluor™ 594	Thermo Fisher Scientific	Cat# A-11016,<br>RRID: AB_2534083
Anti-Goat IgG (H+L) Cross-Adsorbed Donkey Secondary Antibody, Alexa Fluor™ 647	Thermo Fisher Scientific	Cat# A-21447,<br>RRID: AB_2535864
Anti-β-actin (Clone AC-15, mouse)	Sigma-Aldrich	Cat# A1978<br>RRID: AB_476692
Anti-rabbit -IRDye 800CW	LI-COR	LCR-925-32213<br>RRID: AB_2715510
Anti-mouse -IRDye 680RD	LI-COR	Cat# LCR-925-68072<br>RRID: AB_2814912
StemLight™ iPS Cell Reprogramming Antibody Kit:<br>Anti-Oct4A (C30A3)<br>Anti-Sox2 (D6D9)<br>Anti-Nanog (D73G4)	Cell Signalling Technologies	Cat# 9092 C30A3<br>RRID: AB_2799505<br>D6D9;<br>RRID: AB_1904142<br>D73G4;<br>RRID: AB_2798659

Supplemental Methods

Exome sequencing analysis of Proband

TruSight One Clinical Exome sequencing (Illumina, USA) and bioinformatic analysis of 65 retinitis pigmentosa and rod cone dystrophy genes known at the time was performed on patient derived peripheral blood genomic DNA by the Molecular Genetics Department, Sydney Genome Diagnostics at the Sydney Children's Hospitals Network (Westmead). Identified *RPGRIP1* variants were confirmed by Sanger sequencing (Australian Genome Research Facility, AGRF, Westmead, Australia). Final variant classification was determined based on the guidelines from the American College of Medical Genetics and Genomics (Brnich et al., 2018; Pejaver et al., 2022).

Karyotyping

iPSCs were treated with 0.2µg/mL colcemid for 2h at 37°C and then collected and resuspended in 60mM KCl hypotonic solution (Sigma) at 37°C for 20min, followed by addition of fixative (3:1 methanol:glacial acetic acid) at room temperature, 15min. Cells were centrifuged down and resuspended in fixative for 10min, re-centrifuged and resuspended in 2:1 fixative and stored at 4°C overnight. Suspensions were dropped onto 5 slides (2 drops/slide) and air dried, stained with Giemsa for G-banding, before chromosome metaphases were analysed using the Ikaros Karyotyping Platform (MetaSystems). Molecular karyotyping, using genomic DNA isolated from newly derived iPSC lines (passage 8-10), was assessed using Illumina Infinium Global Screening Array-24 v3.0 by the Victorian Clinical Genetic Services (Murdoch Children's Research Institute, Victoria, Australia).

Embryoid body (EB) in vitro trilineage differentiation potential

iPSC cultures were maintained in Gibco Essential 8 (E8) medium (Cat. # A1517001, Life Technologies) on Matrigel (Cat. # 354277, Corning) extracellular matrix and passaged weekly. Early passage cells were cryogenically frozen using CryoStor® CS10 (# 07930, StemCell Technologies) as per the manufacturer's protocol. For EB formation, cell aggregates lifted off from confluent cultures were transferred to non-tissue culture treated dishes containing E8 medium and 10µM Y-27632 (Sigma Aldrich). The next day, EB dishes were replaced with spontaneous differentiation medium: KnockOut Dulbecco's Modified Eagle's Medium, 20% KnockOut serum replacement, 1% non-essential amino acids, 1x penicillin-streptomycin, 1% glutamax and 0.1 mM β-mercaptoethanol (Life Technologies), which was changed again after 3 days. On day 7, EBs were collected and plated onto Matrigel coated wells and cultured a further 7 days in spontaneous differentiation medium prior to cell harvest for RT-qPCR using primers listed in Table S3. Experiments were performed on iPSC cultures with passage numbers >12 prior to initiating retinal differentiations.

Immunohistochemistry (IHC) and image analysis

Retinal organoids were either fixed in 4% PFA at room temperature for 45 minutes, then equilibrated in 20% sucrose overnight at 4°C and embedded in Tissue Freezing Medium (Leica) or embedded immediately after rinsing in dPBS (i.e. unfixed). All frozen blocks were cryosectioned to 14um thickness. For immunostaining, sections were immersed in blocking solution (0.1% fish gelatin, and 0.02% triton X-100 in PBS) at room temperature for at least 1 hour. Primary antibodies diluted in blocking solution were applied to slides overnight at 4°C followed with washing in blocking solution for 5 minutes repeated twice. Incubation with secondary antibodies and DAPI was performed at room temperature for 2 hours in the dark. Slides were washed in blocking solution and then mounted in 70% glycerol. For assessing misfolded protein aggregation, the PROTEOSTAT Aggresome

Detection kit (Enzo Life Sciences, Switzerland) was used as per the manufacturer's protocol. All antibodies used are listed in Table S4. All images were taken on a LSM 880 confocal fluorescence microscope (Zeiss) from the ACRF Telomere Analysis Centre (ATAC) Imaging Facility at CMRI. Z-stack and tile-scan 40x objective imaging captured whole-organoid images for fixed organoid sections. For unfixed organoid sections stained with RPGRIP1, RPGR or CEP290 antibodies, 5-8 63x Airyscan images were taken per organoid.

The ImageJ Colocalization plugin (https://imagej.net/ij/plugins/colocalization.html) combined with the particle count function was used to measure the area (pixel) of RPGR and CEP290 co-staining of each 63x airyscan image and averaged values calculated from 5-8 images per organoid. Zen software (Zeiss) was used for 1) manual enumeration of accumulated cGMP and PROTEOSTAT dye total counts from stained whole-organoid images, 2) manual enumeration of 4-8 areas of defined size were used to calculate average counts per whole organoid image (PRPH2 foci and DAPI), 3) measuring the perimeter length (mm) of the outer limiting membrane (OLM) of each organoid to normalise whole-organoid counts to organoid size, and 4) outlining IS (SOD2) and ONL (DAPI) versus OS photoreceptor regions where integrated intensities of rhodopsin fluorescence staining were measured using ImageJ software.

Western blotting

Protein was extracted from cells lysed in RIPA buffer (Thermo Fisher) and protease inhibitors (Roche) and resolved through NuPAGE 4-12% Bis-Tris protein gels (Thermo Fisher Scientific) prior to transfer onto nitrocellulose membrane. Blots were blocked in 5% skim milk for 1 hour at room temperature followed by overnight incubation with primary antibodies RPGRIP1 (Thermo Fisher Scientific) and b-actin (Sigma Aldrich) diluted in 1% skim milk in 1xTBS-0.1% tween-20. Membranes were washed 3x in 1xTBS-0.1% tween-20 for 15 min each and then incubated with fluorescent secondary antibodies (IRDye 800CW Donkey anti-Rabbit IgG or IRDye 680RD Donkey anti-Mouse IgG, Li-COR) for 1 hour at room temperature. Membranes were washed 3x in 1xTBS-0.1% tween-20 for 15 min and then imaged using the ChemiDoc MP (BioRad) system. Protein bands were quantified by Image Lab 6.0 software. Values were normalised to β-actin levels and the ratio versus Control-1 calculated for each sample.

Gene expression analysis (RT-qPCR and RT-dPCR)

Frozen cell pellets or organoids (3 pooled per sample) were lysed in QIAzol for total RNA extraction using the RNeasy Micro Kit with on-column DNase treatment (Qiagen). Total RNA was converted to cDNA using the Superscript IV 1st Strand System (Life Technologies, Thermo Fisher Scientific) for qPCR reactions set up with 1x SensiMix SYBR (Bioline) and 0.25µM primer pairs (Table S3) using the Rotor-Gene 6000 Cycler system (Qiagen). Relative expression levels ($2^{\Delta CT}$ or $2^{\Delta\Delta CT}$) were normalised to geomean cT values of two housekeeper genes, *HPRT* and *POLR2A*. Gene expression in individual retinal organoids was assessed by RT-digital PCR (dPCR) and the QIAcuity dPCR system. PCR reactions were set up using the QIAcuity EvaGreen PCR kit (Qiagen) with addition of template consisting of diluted cDNA (1:5) transcribed from RNA extracted per organoid. Reactions were loaded onto 24-well nanoplates containing 26K partitions. Plates were run at the following thermocycling conditions: hot start at 95 ^{o}C for 2 min followed by 40 cycles of 95 ^{o}C for 15 sec, 60 ^{o}C for 15 sec and 72 ^{o}C for 15 sec, ending with a cooling down step at 40 ^{o}C for 5 min. Plate imaging was performed on the green channel at 200 ms for 3 min. The QIAcuity Software Suite was used to acquire amplicon concentrations (copies/µL) and expression levels normalised to *HPRT*.

Nanopore analysis of full-length RPGRIP1 cDNA amplicons

Amplification of full-length *RPGRIP1* cDNA was performed using the Platinum™ SuperFi™ II Green PCR Master Mix (Thermo Fisher Scientific), 0.5 µM RPGRIP1 primers (Table S3) and retinal organoid cDNA template. Amplicons were purified using the Wizard SV Gel and PCR Clean-up System (Promega) and at least 500ng of total DNA provided to the Ramaciotti Centre for Genomics (UNSW Sydney, Australia) for targeted Oxford Nanopore sequencing. FASTQ files were analysed by Geneious Prime software using minimap2 aligner tool to compare long reads against the NM_020366.4 *RPGRIP1* reference sequence.

Bulk RNA-sequencing of organoids

Total RNA was extracted from at least 3 retinal organoids pooled per sample using the QIAzol and RNeasy Micro kit (Qiagen). The yield and integrity were determined by Tapestation analysis (Agilent). Library preparation with poly A selection (non-strand specific) (VAHTS® Universal V8 RNA-seq Library Prep Kit for Illumina) and sequencing was performed by Azenta (Suzhou, China) using the Illumina NovaSeq platform at a depth of 20 million 2 x 150bp paired-end reads totalling ~6.0 GB of data generated per sample.

RNA-Seq raw fastq files were quality checked by FastQC (version 0.11.9; https://www.bioinformatics.babraham.ac.uk/projects/fastqc/). Trimming of adapters and quality filtering were then performed by AdapterRemoval (version 2.3.2)(Schubert et al., 2016). Trimmed and quality filtered paired-end reads were aligned to the human GRCh38 reference genome using STAR (version 2.7.9a)(Dobin et al., 2013) with default parameters. Gene expression was quantified by counting the number of reads aligned to each Ensembl gene model using featureCounts (version 2.0.1).(Liao et al., 2014) The Trimmed Mean of M-values (TMM) normalisation method from edgeR package in R (version 3.36.0) was applied to normalise read counts according to library size differences between samples (Robinson et al., 2010).

Differential expression (DE) analysis was performed using the edgeR. Genes with low counts were filtered out (CPM value < 2 in the smallest sample group), and a model matrix was constructed based on the three condition groupings (Control, MS-VUS, and LCA-1). A negative binomial generalized linear model was fitted, and differential expression was assessed using the likelihood ratio test, with *p*-values adjusted for multiple testing using the Benjamini-Hochberg procedure. Statistical significance was considered at *p*-adjusted value < 0.05.

The RPGRIP1 signature was generated by intersecting RPGRIP1-associated gene sets with differentially expressed genes identified from the bulk RNA-seq data. First, using the human C5 ontology gene sets from MSigDB, we identified all pathways that included the gene, RPGRIP1. Among these gene sets, only those that had the terms “EYE”, “RETINAL”, “CILIUM”, and “PHOTORECEPTOR” within their name AND had a gene set size of greater than 10 genes were kept. Second, DE analysis was performed using the bulk RNA-seq data as described above. To account for the differences in genetic background between Control-1/MS-VUS and LCA-1, significant DE genes between Control-1 and MS-VUS were considered. Finally, these two gene sets were intersected to generate the final RPGRIP1 signature set, leading to a total of 181 genes.

To investigate whether the change in gene expression of the *RPGRIP1* signature gene set shows a similar pattern in the pathogenic samples, MS-VUS and LCA-1, relative to the control sample, Control-1, we performed residual analysis based on linear models fitted to the expression data. The linear regression models were fitted using the ordinary least squares method. For each model, the residuals, defined as the differences between the observed and predicted expression values, were extracted.

Single cell RNA-sequencing and analysis

Retinal organoids were dissociated into single cells using the Neurosphere Dissociation Kit (P) (Miltenyi Biotec, Macquarie Park, Australia), as per the manufacturer's protocol, and provided to the Single Cell Analytics Facility at CMRI for portioning into single cells and library construction using the Chromium Next GEM Single cell 3' Gel Bead and Library kit v3.1 (10x Genomics). Libraries were sequenced by Azenta (Suzhou, China) on a single NovaSeq lane at a depth of 200M paired end reads each.

Sequencing results were processed using CellRanger with default parameters (v3.1, 10x Genomics) to extract genomic reads which were aligned against annotated human genome, including the protein and non-coding transcripts (GRCh38, GENCODE v27). The reads with the same cell barcode and unique molecular identifier were collapsed to a unique transcript, generating the count matrix where columns correspond to single cells and rows correspond to transcripts. To remove potentially empty droplets with ambient RNA, the emptyDrops function from the DropletUtils package was used (Lun et al., 2019). Droplets with significantly non-ambient profiles were called at a false discovery rate of 1%, applying the Benjamini-Hochberg method for multiple testing. To remove suboptimal cells, cells with fewer than 1000 genes expressed, more than 7000 genes expressed or greater than 20% of mitochondrial gene expression were removed. DoubletFinder was used to remove potential doublets or multiplets from each biological batch at a threshold of 7.5% (McGinnis et al., 2019).

Classification of retinal single cells was performed following the Seurat framework (Butler et al., 2018). The count matrices from our in-house data and the human retinal reference (Swamy et al., 2021) were normalised and log-transformed using the NormalizeData() function. Variable genes were identified using the FindVariableFeatures() function and the variance-stabilising transformation method, and the top 2000 variable genes were retained for downstream analysis. Using the integrated human reference, we performed single-cell referencing mapping to perform cell-type label transfer unto our query datasets (Cowan et al., 2020). Transfer anchors were identified between the reference and query datasets by employing the FindTransferAnchors() function using the first 30 PCs of the reference data. Then TransferData() function was applied to classify the cells in the query datasets based on the reference data. Any mislabels in the cell type predictions were corrected using the scReClassify framework as described previously (Kim et al., 2019) to generate our final high-quality annotations. To further investigate the heterogeneity of Rod cells, we performed an unsupervised clustering of the Rod population to identify subpopulations. We performed Louvain clustering using igraph's cluster_louvain() function on the shared nearest neighbour graph (k = 10) constructed on the first 50 PCs. The principal components were quantified using negative binomial GLM-PCA (Townes et al., 2019). Sub-clusters between datasets were harmonized by performing hierarchical clustering on the Cepo statistics derived for each cluster where k = 3.

To derive the differential stability (DS) statistics, a gene-wise measure of cellular identity from single-cell RNA-seq data was calculated using the Cepo package (Kim et al., 2021). The count matrix of cell-gene variables was first log-transformed and normalized using the logNormCounts function from the scater package (McCarthy et al., 2017). Then Cepo was subsequently applied on the transformed and normalized data from each batch to quantify cell identity gene statistics for each major cell type based on the differential stability metric.

Gene set enrichment analysis

Gene set enrichment analysis (GSEA) was conducted using either the differential expression statistics or differential stability statistics. The ranked list of genes, based on either the log fold changes from

the differential expression analysis or the differential stability statistics, was used as input. GSEA was performed using the fgsea package in R (Korotkevich et al., 2019) with gene sets derived from the MSigDB database (Liberzon et al., 2015). The analysis identified significantly enriched pathways, with *p*-values adjusted for multiple comparisons using the Benjamini-Hochberg procedure. Enrichment plots were generated to visualize key pathways using the ggplot R package (ggplot2: Elegant Graphics for Data Analysis).

RPGRIP1 AAV construct creation and organoid transductions

An AAV2 construct was kindly supplied by Dr Sharon Cunningham (CMRI, Westmead, Australia) and modified for this study as follows. Restriction enzymes PacI and NotI (NEB, Victoria, Australia) were used to excise the original promoter sequence which was replaced with a segment of the human rhodopsin kinase (*GRK1*) promoter (-112 to +87, GenBank: AY327580.1) to drive transgene expression specifically in photoreceptor cells (Beltran et al., 2010). *RPGRIP1* cDNA encoding isoform 1 (NM_020366.4), with Kozak sequence directly before the ATG start codon and a c-terminal-HA tag, was synthesised by Genscript Biotech (Hong Kong, China) and cloned into the construct in place of the original transgene flanked with BsiWI and SalI restriction enzyme sites (NEB, Victoria, Australia). The expression cassette in the final construct produced was verified by Sanger sequencing (AGRF). An AAV construct containing an GRK1-P2A-GFP expression cassette was also created for control transductions. All plasmid propagations were performed using One Shot Stbl3 competent cells (Invitrogen). AAV vector production using AAV serotype 7m8 was performed by the Vectorology Facility at CMRI (Westmead, Australia). Viral particles were purified by CsCl ultracentrifugation and titres determined by ddPCR (Biorad). Day 130-150 retinal organoids were transduced individually in 96-well plate wells using $1x10^{11}$ vg per organoid in 50uL of ALT90 medium for 8 hours, followed by media topped-up to 100 µL and then 200 µL after 24 hours. On the fourth day, complete media change was performed, and organoids were maintained as normal until collected for analysis.

Supplemental references

Beltran, W.A., Boye, S.L., Boye, S.E., Chiodo, V.A., Lewin, A.S., Hauswirth, W.W., and Aguirre, G.D. (2010). rAAV2/5 gene-targeting to rods:dose-dependent efficiency and complications associated with different promoters. Gene therapy *17*, 1162-1174. 10.1038/gt.2010.56.

Brnich, S.E., Rivera-Munoz, E.A., and Berg, J.S. (2018). Quantifying the potential of functional evidence to reclassify variants of uncertain significance in the categorical and Bayesian interpretation frameworks. Hum Mutat *39*, 1531-1541. 10.1002/humu.23609.

Butler, A., Hoffman, P., Smibert, P., Papalexi, E., and Satija, R. (2018). Integrating single-cell transcriptomic data across different conditions, technologies, and species. Nat Biotechnol *36*, 411-420. 10.1038/nbt.4096.

Cowan, C.S., Renner, M., De Gennaro, M., Gross-Scherf, B., Goldblum, D., Hou, Y., Munz, M., Rodrigues, T.M., Krol, J., Szikra, T., et al. (2020). Cell Types of the Human Retina and Its Organoids at Single-Cell Resolution. Cell *182*, 1623-1640 e1634. 10.1016/j.cell.2020.08.013.

Dobin, A., Davis, C.A., Schlesinger, F., Drenkow, J., Zaleski, C., Jha, S., Batut, P., Chaisson, M., and Gingeras, T.R. (2013). STAR: ultrafast universal RNA-seq aligner. Bioinformatics *29*, 15-21. 10.1093/bioinformatics/bts635.

ggplot2: Elegant Graphics for Data Analysis. (SpringerLink [Internet]. [cited 2024 Oct 13]. Available from: https://link.springer.com/book/10.1007/978-3-319-24277-4).

Kim, H.J., Wang, K., Chen, C., Lin, Y., Tam, P.P.L., Lin, D.M., Yang, J.Y.H., and Yang, P. (2021). Uncovering cell identity through differential stability with Cepo. Nat Comput Sci *1*, 784-790. 10.1038/s43588-021-00172-2.

Kim, T., Lo, K., Geddes, T.A., Kim, H.J., Yang, J.Y.H., and Yang, P. (2019). scReClassify: post hoc cell type classification of single-cell rNA-seq data. BMC Genomics *20*, 913. 10.1186/s12864-019-6305-x.

Korotkevich, G., Sukhov, V., and Sergushichev, A. (2019). Fast gene set enrichment analysis [Internet]. bioRxiv; [cited 2024 Oct 2013]. p. 060012. Available from: https://www.biorxiv.org/content/060010.061101/060012v060012.

Liao, Y., Smyth, G.K., and Shi, W. (2014). featureCounts: an efficient general purpose program for assigning sequence reads to genomic features. Bioinformatics *30*, 923-930. 10.1093/bioinformatics/btt656.

Liberzon, A., Birger, C., Thorvaldsdottir, H., Ghandi, M., Mesirov, J.P., and Tamayo, P. (2015). The Molecular Signatures Database (MSigDB) hallmark gene set collection. Cell Syst *1*, 417-425. 10.1016/j.cels.2015.12.004.

Lun, A.T.L., Riesenfeld, S., Andrews, T., Dao, T.P., Gomes, T., participants in the 1st Human Cell Atlas, J., and Marioni, J.C. (2019). EmptyDrops: distinguishing cells from empty droplets in droplet-based single-cell RNA sequencing data. Genome Biol *20*, 63. 10.1186/s13059-019-1662-y.

McCarthy, D.J., Campbell, K.R., Lun, A.T., and Wills, Q.F. (2017). Scater: pre-processing, quality control, normalization and visualization of single-cell RNA-seq data in R. Bioinformatics *33*, 1179-1186. 10.1093/bioinformatics/btw777.

McGinnis, C.S., Murrow, L.M., and Gartner, Z.J. (2019). DoubletFinder: Doublet Detection in Single-Cell RNA Sequencing Data Using Artificial Nearest Neighbors. Cell Syst *8*, 329-337 e324. 10.1016/j.cels.2019.03.003.

Nash, B.M., Loi, T.H., Fernando, M., Sabri, A., Robinson, J., Cheng, A., Eamegdool, S.S., Farnsworth, E., Bennetts, B., Grigg, J.R., et al. (2021). Evaluation for Retinal Therapy for RPE65 Variation Assessed in hiPSC Retinal Pigment Epithelial Cells. Stem Cells Int *2021*, 4536382. 10.1155/2021/4536382.

Pejaver, V., Byrne, A.B., Feng, B.J., Pagel, K.A., Mooney, S.D., Karchin, R., O'Donnell-Luria, A., Harrison, S.M., Tavtigian, S.V., Greenblatt, M.S., et al. (2022). Calibration of computational tools for missense variant pathogenicity classification and ClinGen recommendations for PP3/BP4 criteria. Am J Hum Genet *109*, 2163-2177. 10.1016/j.ajhg.2022.10.013.

Robinson, M.D., McCarthy, D.J., and Smyth, G.K. (2010). edgeR: a Bioconductor package for differential expression analysis of digital gene expression data. Bioinformatics *26*, 139-140. 10.1093/bioinformatics/btp616.

Schubert, M., Lindgreen, S., and Orlando, L. (2016). AdapterRemoval v2: rapid adapter trimming, identification, and read merging. BMC Res Notes *9*, 88. 10.1186/s13104-016-1900-2.

Swamy, V.S., Fufa, T.D., Hufnagel, R.B., and McGaughey, D.M. (2021). Building the mega single-cell transcriptome ocular meta-atlas. Gigascience *10*. 10.1093/gigascience/giab061.

Townes, F.W., Hicks, S.C., Aryee, M.J., and Irizarry, R.A. (2019). Feature selection and dimension reduction for single-cell RNA-Seq based on a multinomial model. Genome Biol *20*, 295. 10.1186/s13059-019-1861-6.
